# A Pan-Cancer Catalogue of Cancer Driver Protein Interaction Interfaces

**DOI:** 10.1371/journal.pcbi.1004518

**Published:** 2015-10-20

**Authors:** Eduard Porta-Pardo, Luz Garcia-Alonso, Thomas Hrabe, Joaquin Dopazo, Adam Godzik

**Affiliations:** 1 Bioinformatics and Systems Biology Program, Sanford-Burnham Medical Research Institute, La Jolla, California, United States of America; 2 European Bioinformatics Institute (EMBL-EBI), Wellcome Trust Genome Campus, Cambridge, United Kingdom; 3 Computational Genomics Department, Centro de Investigación Príncipe Felipe (CIPF), Valencia, Spain; 4 Functional Genomics Node, (INB) at CIPF, Valencia, Spain; 5 Bioinformatics of Rare Diseases (BIER), CIBER de Enfermedades Raras (CIBERER), Valencia, Spain; National Cancer Institute, United States of America and Tel Aviv University, Israel, UNITED STATES

## Abstract

Despite their importance in maintaining the integrity of all cellular pathways, the role of mutations on protein-protein interaction (PPI) interfaces as cancer drivers has not been systematically studied. Here we analyzed the mutation patterns of the PPI interfaces from 10,028 proteins in a pan-cancer cohort of 5,989 tumors from 23 projects of The Cancer Genome Atlas (TCGA) to find interfaces enriched in somatic missense mutations. To that end we use e-Driver, an algorithm to analyze the mutation distribution of specific protein functional regions. We identified 103 PPI interfaces enriched in somatic cancer mutations. 32 of these interfaces are found in proteins coded by known cancer driver genes. The remaining 71 interfaces are found in proteins that have not been previously identified as cancer drivers even that, in most cases, there is an extensive literature suggesting they play an important role in cancer. Finally, we integrate these findings with clinical information to show how tumors apparently driven by the same gene have different behaviors, including patient outcomes, depending on which specific interfaces are mutated.

## Author Summary

Until now, most efforts in cancer genomics have focused on identifying genes and pathways driving tumor development. Although this has been unquestionably a success, as evidenced by the fact that we now have an extensive catalogue of cancer driver genes and pathways, there is still a poor understanding of why patients with the same affected driver genes may have different disease outcomes or drug responses. This is precisely the aim of this work-to show how by considering proteins as multifunctional factories instead of monolithic black boxes, it is possible to identify novel cancer driver genes and propose molecular hypotheses to explain such heterogeneity. To that end we have mapped the mutation profiles of 5,989 cancer patients from TCGA to more than 10,000 protein structures, leading us to identify 103 protein interaction interfaces enriched in somatic mutations. Finally, we have integrated clinical annotations as well as proteomics data to show how tumors apparently driven by the same gene can display different behaviors, including patient outcomes, depending on which specific interfaces are mutated.

## Introduction

Cancer patients are extremely heterogeneous in their response to treatments and disease outcomes. The first step towards the understanding of this variability was the identification of the multitude of genes that cause cancer, the so-called cancer driver genes[1]. In that sense, the completion of The Cancer Genome Atlas (TCGA) and other large-scale cancer genomics projects was a watershed event, as it provided the critical mass of data needed to identify driver alterations in most types of cancers[2–15]. Moreover, cancer types that previously were thought to represent homogenous diseases were found to constitute different subtypes with different outcomes depending on the specific driver events in each patient[16]. Since the start of the TCGA project, the catalogue of cancer driver genes has increased and become more accurate[17] thanks not only to the data generated by the project itself, but also to the development of multiple, complimentary algorithms that search for cancer driver genes using different approaches. For example, some of these methods identify cancer drivers by searching for genes with higher than expected mutation rates[18,19], whereas others identify genes that tend to accumulate damaging mutations[20] or contain regions with an unusually high proportion of mutations[21,22].

Nevertheless, the catalogue of cancer driver genes is far from complete and, because of extreme mutation diversity, it is hard to extend it by simply increasing the size of the datasets[19]. A complementary approach towards that goal is to use methods that integrate cancer mutation profiles with other types of biological knowledge to increase the statistical power of the analysis. For example, by integrating the information on the mutation profile of cancer patients with biological networks we can identify pathways and protein complexes that are recurrently mutated in cancer and are, therefore, likely drivers[23]. Note that these complexes can only be identified as drivers when adding the signals of all the components, because each individual protein is rarely mutated and, thus, missed by standard gene-centric approaches. In fact, a recent paper describes the crucial role played by the network topology in the final phenotypic effect of apparently deleterious mutations[24].

Similarly, we can include information on the structure of the protein coded by genes being analyzed to check enrichment in cancer mutations in specific structural regions[22,25–27]. The underlying idea for this approach is that genes (and the proteins they encode) are not monolithic entities, but instead consist of different regions usually responsible for different functions. In that context, it is possible that a given protein acts as a driver only when a specific region is mutated. This idea can be exploited to identify cancer driver genes by analyzing the distribution of mutations within a gene and looking for regions with unusually high mutation rates. Such fine grain approaches are not only capable of finding novel cancer drivers, but they also can help explain some of the variability between tumors or cancer cell lines apparently driven by the same gene[28]. We have previously developed an algorithm, e-Driver, which exploits this feature to identify cancer driver genes based on linear annotations of biological regions such as protein domains[22]. Despite encouraging results, the algorithm still had some limitations, as many structural features, like protein interaction interfaces, may be discontinuous at the sequence level and, hence, can not be analyzed without explicit use of protein structure information.

Here we introduce an extended version of e-Driver that uses information on three-dimensional structures of the mutated proteins to identify specific structural features. Then, the algorithm analyzes whether these features are enriched in cancer somatic mutations and, therefore, are candidate driver genes. While technically the analysis can be applied to any structural feature or region, here we focus our attention on protein-protein interaction (PPI) interfaces. Many known cancer driver genes are located in critical regions of the PPI network (interactome), usually in network hubs or bottlenecks[29], warranting closer investigation of interaction interfaces. Moreover, while it is known that many cancer somatic alterations alter PPI interfaces, either destroying existing interactions or creating new ones[30–32], this question has never been systematically analyzed across all known cancer somatic mutations with the specific goal of finding protein interfaces enriched in cancer mutations.

Our analysis identified PPI interfaces enriched in somatic cancer mutations in a total of 103 genes (interface driver genes). Thirty-two of these are well-known cancer driver genes, which are strongly enriched in somatic missense mutations and were previously identified using other algorithms and approaches. We also found that interface driver genes have an unusually high number of interactions in all known PPI interaction network models. This effect is especially pronounced for the 32 known cancer drivers, not only when compared to the rest of the genes in the interactome, but also when compared to non-interface cancer driver genes. The role of the remaining 71 genes as cancer drivers will obviously have to be verified experimentally, though we find some attributes as well as literature links that, albeit indirectly, support the prediction of them being cancer drivers. Interestingly, many of the new putative “interface driver genes” are involved in the immune response, particularly in HLA-like and complement systems. The role of the immune system in cancer treatment and evolution is gaining increasing attention[33,34] and our results provide new details regarding which interactions seem to be most affected by somatic mutations. Finally we show how, in many cases, depending on which interface or protein region is altered, tumors apparently driven by the same cancer gene might have radically different behaviors and patient outcomes.

## Results

### e-Driver reveals driver interfaces

We assembled a data set consisting of 5,989 tumors from 23 cancer types from The Cancer Genome Atlas[35] (Table A in S1 Table). The number of samples per tumor type ranged from 56 for uterine carcinosarcoma, to 975 for breast adenocarcinoma (Fig A in S1 Text). Consistent with previous reports[36], the average number of missense mutations per sample is highly variable among cancer types (Fig B in S1 Text), with melanoma having the highest (429 missense mutations per sample) and thyroid carcinoma the lowest (11 missense mutations per sample).

**Fig 1:**
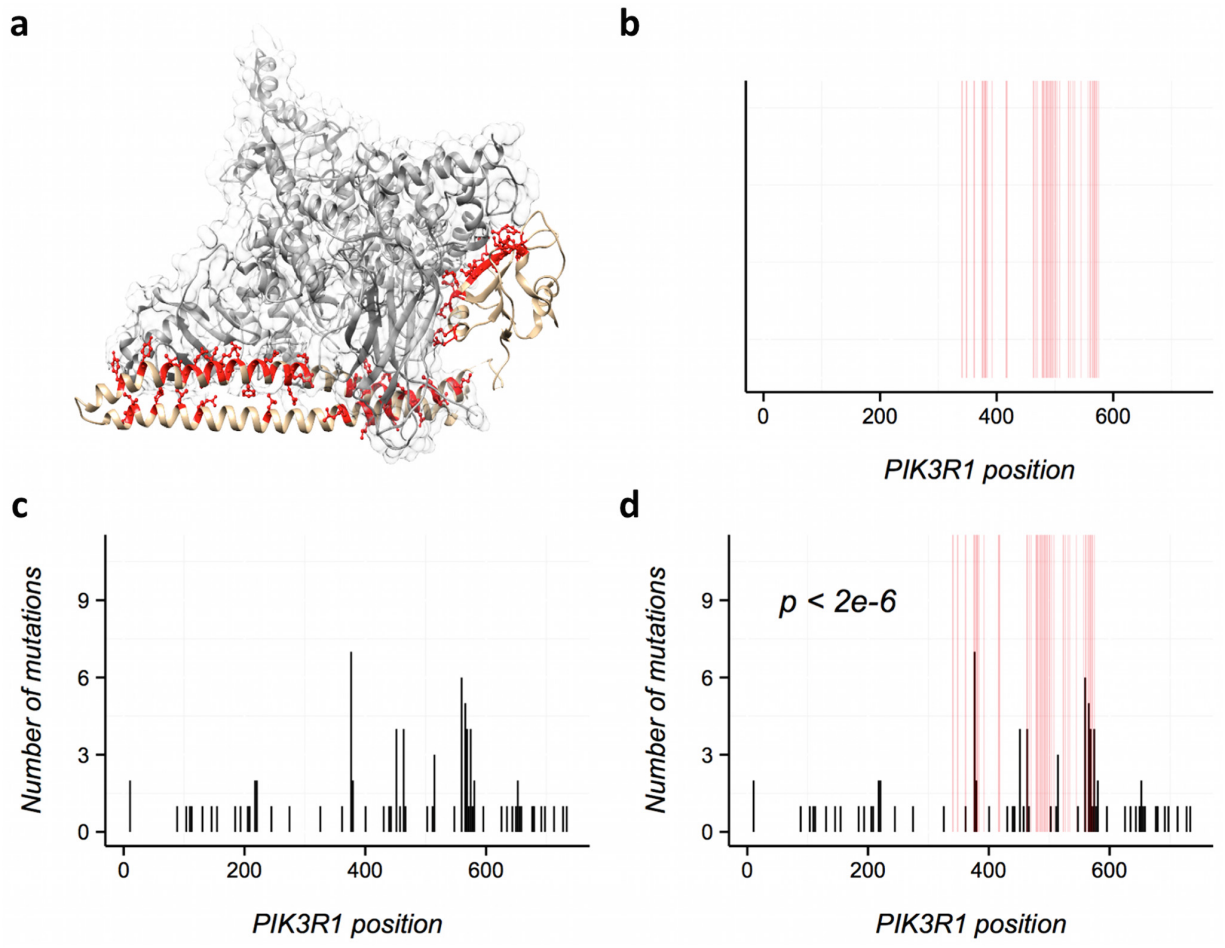
Using e-Driver to analyze PIK3R1 PPI interfaces. a) The PDB coordinate set 3HMM contains 2 chains, A (a region of PIK3CA, shown in gray) and B (a region of PIK3R1, shown in brown). Residues from PIK3R1 colored in red make the interaction interface with PIK3CA. b) Using BLAST we then map these residues to the corresponding ENSEMBL protein, define a PPI interface (shown in red). Note that the interface is not continuous in sequence. c) Distribution of mutations in PIK3R1 across all cancer types. d) Using e-Driver we can identify the interface between PIK3R1 and PIK3CA as strongly enriched in missense somatic mutations (p < 2e-6).

We then compiled a list of currently known, high-confidence PPI interfaces using 18,651 protein structures downloaded from PDB (Online methods). In short, we defined a PPI interface as the set of residues from a given chain that are within 5 angstroms of any residue from a different chain in the same set of PDB coordinates (Fig 1a). We identified 122,326 different PPI interfaces between 70,199 PDB chains (Online methods). Finally, we used BLAST to map the residues from the PDB datasets to gene sequences in the ENSEMBL human genome. Overall we mapped the PDB coordinates to 11,154 protein isoforms in 10,028 different human genes. The mapping covers roughly 30% of the human proteome (measured per amino acid), with 6% of the proteome being mapped to at least one PPI interface.

Mutations from all cancer datasets (n = 868,508) are distributed randomly across the proteome, with approximately 30% of mutations (n = 285,942) being in regions mapped to structures and around 6% in PPI interfaces (n = 67,174). However, in the case of known cancer driver genes [1,17], regions covered by structures have between 20% and 60% more missense mutations than expected by chance (Figs C and D in S1 Text), as we could map, on average, 40% of all mutations in known driver genes to a structurally solved region. This enrichment, while variable and dependent on the cancer type, is even higher, between two and three-fold, in regions involved in the PPI interfaces. For example, PPI interfaces from cancer driver genes in breast adenocarcinoma, glioblastoma, lower grade glioma rectal adenocarcinoma or uterine carcinosarcoma (Fig D in S1 Text) have more than three times as many mutations as would be expected by chance. These results strongly suggest that, indeed, mutations in PPI interfaces play key roles in carcinogenesis.

To analyze the potential role of mutations in other proteins in cancer development, we used e-Driver to analyze individual PPI interfaces for all human proteins in each of the 23 individual cancer projects, as well as in the Pan-cancer dataset consisting of the combination of all of them. Briefly, e-Driver compares the observed number of mutations in a specific protein region with the expected value based on the ratio between the length of the given region and the length of the protein. We had previously used e-Driver to analyze the distribution of cancer somatic mutations in PFAM domains and intrinsically disordered regions and showed, for example, that different domains in the same protein can drive different types of cancer[22]. Here, we adapted e-Driver to analyze features that are discontinuous along the protein sequence, such as PPI interfaces identified from 3D structures of protein complexes. The whole process is exemplified in Fig 1 for PIK3R1 and its interaction interface with PIK3CA.

**Fig 2:**
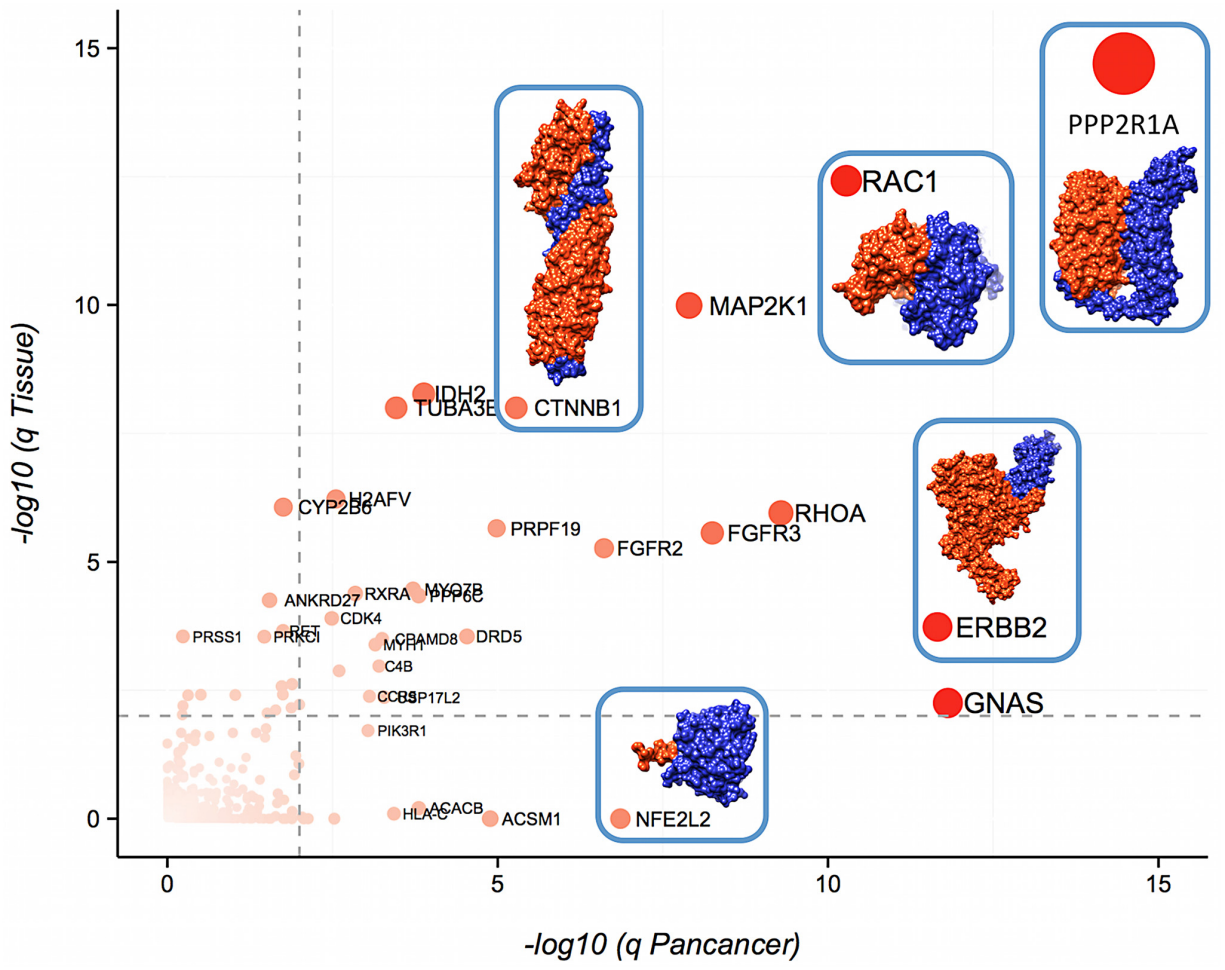
Genes with driver interfaces. X,Y coordinates reflect the q value (FDR) in the Pancancer analysis and their lowest q value in all 23 project-specific analysis, respectively. Gray dashed lines are located at 0.01 FDR for reference. Dots are colored, sized and labeled according to their FDR: genes with an FDR of 1 are colored in white, are smaller, and have no label whereas genes with lower FDRs are redder, bigger, and labeled. Note that there are 8 genes that are not in the plot because their FDR value was too small (FDR < 1e-15): *TP53*, *EGFR*, *KRAS*, *HRAS*, *NRAS*, *RPSAP58*, *PIK3CA* and *UBBP4*. Some driver interfaces are also illustrated in the plot, with the chain belonging to the gene with the driver interface colored in orange and the interacting chain colored in blue. The PDB coordinates used are 3IFQ for CTNNB1, 1S78 for ERBB2, 2FLU for NFE2L2, 3B13 for RAC1 and 3FGA for PP2R1A.

**Fig 3:**
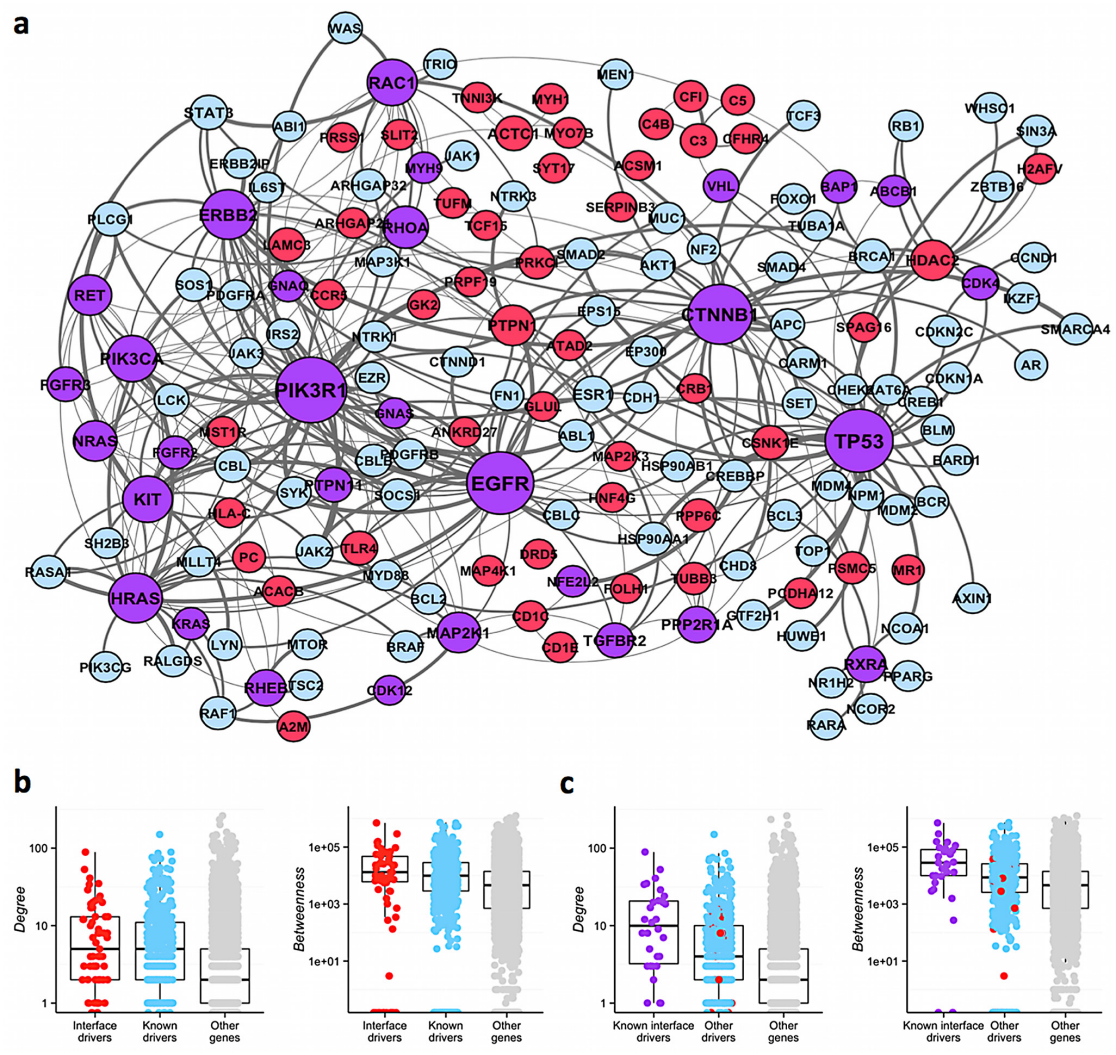
Network properties of interface-driver genes. a) Most genes identified by e-Driver (shown in red) cluster together with other cancer driver genes (shown in light blue). Genes are sized according to their number of interactions (genes with more interactions are bigger and *vice versa*). Note that genes identified by e-Driver that are also known drivers (shown in purple) tend to have many interactions with other cancer genes. Edges are weighted according to the number of networks where they can be identified, with thicker edges being found in more networks, and *vice versa*. b) Interface driver genes have similar network properties when compared to known cancer driver genes. Both groups of genes have higher degree (left) and betweenness (right) than the rest of the genes in the genome. c) Known drivers identified by e-Driver (in purple) have higher degree and betweenness than other drivers (known-in blue- or predicted-in red-) or the rest of the genes in the genome (shown in light gray). Statistics in panels (b) and (c) are based on network properties from the network “Vidal merged”. The statistics for the all networks are in Tables AI and AJ in S1 Table.

We identified a total of 103 interface driver genes in either one of the cancer projects or in the Pan-cancer analysis (FDR < 0.01, Figs 2, 3 and Tables B-Z in S1 Table). There is significant overlap between the genes identified in this analysis and lists of known cancer genes. For example, 32 interface driver genes (31%) are included in either a list of high-confidence driver genes derived from previous analyses of TCGA data[17] or are part of the Cancer Gene Census[1] (p < 1e-10, odds ratio 9, when only taking into account genes with structurally solved regions). To further validate our findings we repeated the analysis using the PPI interfaces from Interactome3D[37]. While the pipeline used to define the interfaces in Interactome3D is different than the one that we used and the coverage is lower, the resulting list of driver interface genes is very similar (Figs E-G in S1 Text and Table AE in S1 Table), supporting the robustness of our analysis.

Note that, as expected, there are many known cancer driver genes (n = 433) that are not picked up by our analysis. These genes might not have been identified either because their mechanism of action does not involve perturbing specific PPI interface, but also because we currently may not have a structure with a PPI interface to match to them and, thus, they were not included our analysis. For example, we can map only 40% of all the mutations in known driver genes to 3D structure models. Many of the remaining 60% of mutations might also be altering interactions, but we will not know that until we increase the structural coverage of the human proteome.

Some of the driver interfaces identified here contain known cancer hotspots. For example, *NFE2L2*, a gene involved in cancer progression and drug resistance, is usually activated by mutations that disrupt the interaction with its repressor *KEAP1*. We mapped 36 mutations from *NFE2L2* to the structure showing its interaction with its repressor *KEAP1* (PDB 2FLU, shown in Fig 2). In agreement with previous observations[15], all but two of the mutations (94%) in *NFE2L2* involve interface residues, likely disrupting the interaction between the two proteins and activating *NFE2L2*.

Our results also highlight similarities and differences across related driver genes. For example, receptor tyrosine kinases, particularly members of the ERBB and FGFR families, are mutated in many cancers and frequently act as drivers. We found two ERBB proteins, ERBB2 and EGFR, among the interface driver genes. These two proteins are both strongly enriched in mutations in their dimerization interfaces, while the ligand-binding region is rarely mutated (Fig N in S1 Text). We also identified two proteins from the FGFR family: FGFR2 and FGFR3. Again, these two proteins have similar mutation profiles, with both proteins having most of their missense mutations in the region that interacts with the ligand, while leaving the dimerization interface intact. This, however, contrasts with the mutation pattern of the ERBB receptors, where, as we have explained, the ligand-binding region is rarely mutated. Since some of the most successful therapeutic antibodies against EGFR target the dimerization interface identified by our method, it is possible that antibodies against FGF receptors need to target the ligand-binding region in order to be successful[38].

Next we analyzed the 71 interface driver genes that are currently not classified as cancer drivers to determine their potential role in cancer. We found several results supporting our hypothesis that these genes can be cancer drivers. For example, many genes in our new-driver predictions are close network neighbors of known cancer drivers (Fig L and M in S1 Text). A subset of them can also be identified by other established methods (such as OncodriveFM[20] or OncodriveCLUST[21], Tables AC and AD in S1 Table). Furthermore, in several cases there is extensive literature and biological evidence supporting this hypothesis. This is the case, for example, for ARGHAP21. This protein is a small Rho GTPase that is suspected to play a role in epithelial-mesenchymal transition[39] and interacts, probably through the interface identified by e-Driver, with the known oncogene ARHGAP26.

Another subset of these 71 potential new cancer driver genes has functions related to immunity. Given the growing body of evidence showing that the immune system plays a key role in cancer progression and patients outcomes[34,40], we analyzed these interfaces in more detail to try to find novel insights about the interplay between tumors and immune cells. For example, a recent pan-cancer analysis identified a subnetwork of proteins around HLA class I proteins as being recurrently mutated in cancer[23]. Our analysis also identified several antigen-presenting molecules as potential cancer drivers, including one class I (HLA-C), one class II (HLA-DRB1), and three HLA-like proteins (CD1C, CD1E and MR1). Note also that HLA-C has been recently identified as a likely driver in head and neck cancer[15]. Another interesting group of immune-related proteins identified in our analysis include several elements of the complement cascade (C3, C4B and C5) or complement regulators and inhibitors (CFHR4, CFI and CPAMD8). The complement molecules C3 and C4 have been previously associated with cancer progression and activation of PI3K signaling[41], whereas C5a is suspected to inhibit CD8 lymphocytes and natural killer (NK) cells, a subset of immune cells involved in the immune response towards tumors [42]. Our analysis with e-Driver not only supports the role of these proteins in cancer, but also suggests a specific mechanism for that role.

### Interface driver genes are network hubs

Cancer driver genes are known to occupy critical positions in the interactome, as well as having more interactions and higher betweenness than the average gene[29]. Since our method identifies additional cancer driver genes, we hypothesized that they would have similar network positions as known cancer drivers. To test this hypothesis, we measured the degree and betweenness centrality of the interface driver genes in 16 different protein interaction and functional networks from 7 different sources (Fig 3 and Figs H-K in S1 Text). In all but one of the networks interface driver genes correlated with higher degrees even after correcting by confounding variables such as number of publications citing the gene[43], whether the gene had a PDB structure or not, or if the gene is a known driver (Table AF in S1 Table). Interface driver genes also correlated with higher betweenness in all but 4 of the networks, after correcting by all the aforementioned variables. Remarkably, while interface driver genes have similar network properties to known cancer drivers (Fig 3b, Table AI in S1 Table), genes that belong to both groups (i.e. known drivers with interfaces enriched in somatic mutations) are located in even more critical positions of the network (Fig 3c, Table AJ in S1 Table). These results are consistent with the hypothesis that the main driver mechanism of the interface driver genes, particularly those with strong driver signatures that are picked by multiple methods, is the alteration of the PPI interfaces and the interactions they mediate.

### Consequences of mutations in driver interfaces

Even if the genes that we identified have more mutations than expected in some of their PPI interfaces, there are tumor samples with mutations in other regions of the same genes. With that in mind, we wondered if there are consistent differences between cancer samples belonging to each of these two groups. To explore this issue, we first used proteomics data[44] and compared the expression levels of different proteins in tumors with mutations in the predicted driver interfaces to that of tumors with mutations in other regions of the same gene. To limit the impact of intrinsic tissue-variability in the protein expression levels, we limited our analysis to tissue-specific driver interfaces.

**Fig 4:**
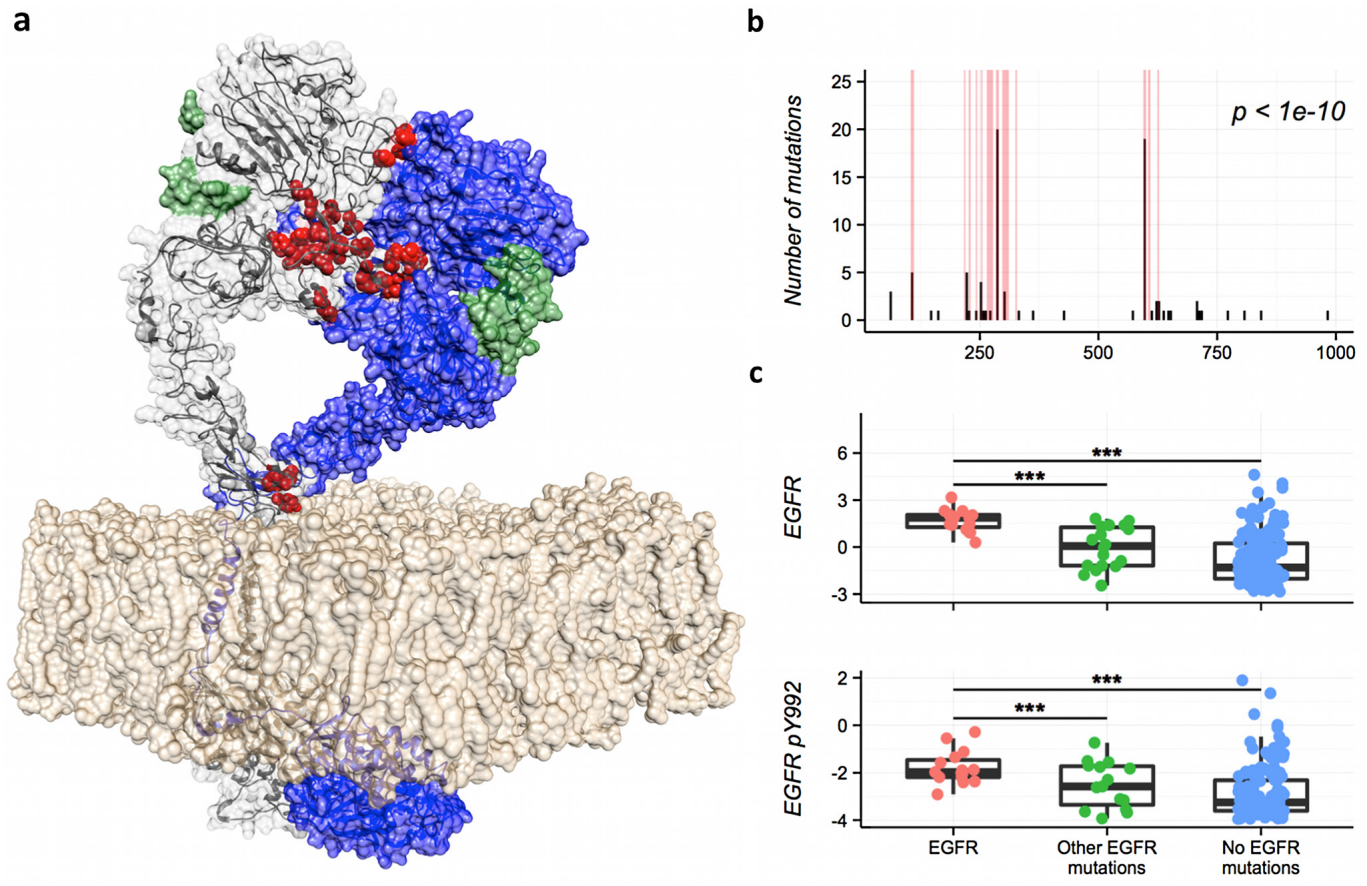
Protein expression changes induced by mutations in EGFR dimerization interface. a) Structure showing an EGFR dimer in active conformation (based on[78]). The residues involved in the EGFR-EGFR interaction for the left EGFR molecule are shown in red, the other EGFR protein is shown in blue, the two EGF ligands are shown in green and the lipid bilayer in brown. b) Histogram showing that most glioblastoma mutations (black bars) in EGFR are located in its dimerization interface (red bars). c) Protein expression in different glioblastoma populations. Patients with mutations in EGFR’s dimerization interface (shown in red) have higher levels of EGFR and phosphorylated EGFR proteins than those with other or no EGFR mutations (shown in green and blue respectively).

Though we could not analyze most of the interfaces due to lack of statistical power (there were not enough samples with proteomics data in both groups), we did find some interface-specific protein changes. For example, glioblastoma samples with mutations in EGFR’s dimerization interface have higher levels of both EGFR and phosphorylated EGFR (Y992 and Y1173) proteins than patients with other EGFR mutations (Fig 4), suggesting that EGFR signaling is stronger in these patients. Note that these results also agree with the hypothesis that the main molecular mechanism driving cancer in these genes is the disruption of certain interactions, as cancer cells have different signaling levels depending on whether the gene is mutated in the identified driver interfaces or in another region.

**Fig 5:**
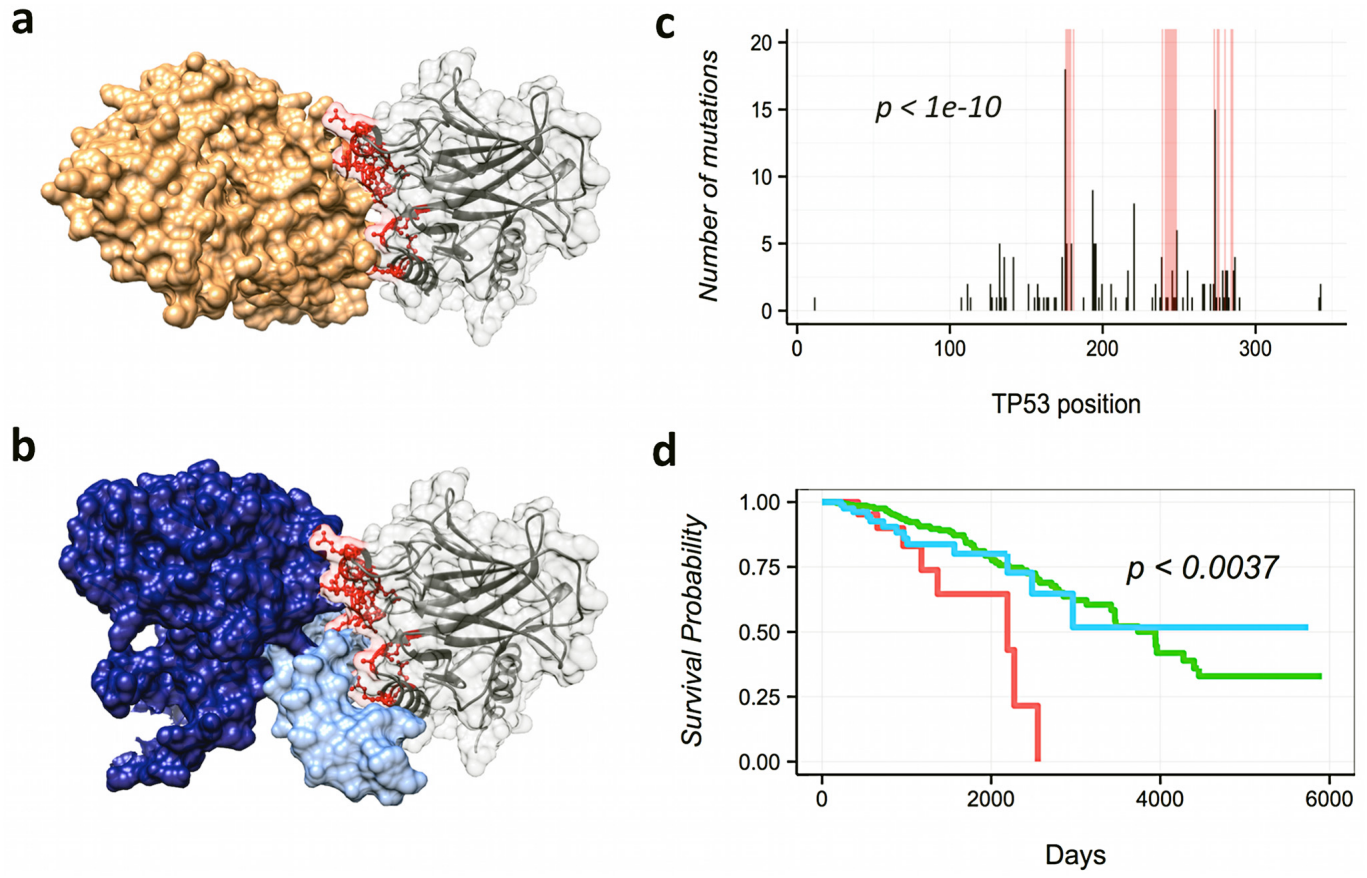
Mutations in TP53 interface predict patient's outcomes. a) Our analysis identified the interface between TP53 and SV40 (shown in gray and orange respectively) as strongly enriched in mutations (panel c). b) This interface is the same as the one that TP53 uses to interact with other TP53 molecules (shown in dark blue) and DNA (shown in light blue). d) Breast cancer patients with mutations in this interface (shown in red) have worse outcomes than those with other or no TP53 mutations (shown in blue and red respectively).

Another example of interface-specific protein expression changes comes from TP53 and its interface with SV40 (Fig 5). Note that this interface is the same as the one that TP53 uses to dimerize and bind to DNA (Fig 5b). Patients from eight different cancer types (bladder, breast, colon, endometrial, glioma, stomach, lung and head and neck) with mutations in this interface had significantly higher levels of TP53 protein than those with other or no TP53 mutations (Fig H in S1 Text). Moreover, patients with breast cancer had significantly worse outcomes (Fig 5d and Tables AG and AH in S1 Table), suggesting that these mutations are more aggressive than other mutations in TP53 and that maybe different therapeutic approaches are needed in these cases. The association was observed also after correcting by patient age, though because of insufficient statistics we were not able to test other potentially confounding variables such as ER status. Note that traditional gene-centric analyses or the previous version of e-Driver cannot find these differences among patient subpopulations.

## Discussion

In this manuscript we have explored the role of missense mutations in PPI interfaces as cancer driver events using our e-Driver algorithm and the mutation profiles of 5,989 tumor samples from 23 different cancer types. Though the interaction interfaces of many cancer driver genes have been studied before[45,46], this is the first time that three-dimensional protein features, such as PPI interfaces, have been systematically used to identify driver genes across large cancer datasets. Previous large scale analyses are either limited to linear features[22,24,25,47], or are not based on known functional regions in three-dimensional structures but, instead, identify *de novo* three-dimensional clusters of mutations[26]. Our analysis identified several driver PPI interfaces in known cancer driver genes, such as TP53, HRAS, PIK3CA or EGFR, proving that our method can find relevant genes and that alteration of interaction interfaces is a common pathogenic mechanism of cancer somatic mutations. In fact, we found that cancer driver genes, as a group, are strongly enriched (over two-fold in most cancer types, and over three-fold in some cases) in mutations in their PPI interfaces. Moreover, there is a strong correlation between the fact that a cancer driver gene is recurrently mutated on its PPI interfaces and how critical it is to the stability of the interactome in terms of both number of interactions and network betweenness. We also identified a series of driver interfaces in genes that are currently not known as cancer drivers. Some of these genes interact with known cancer drivers or are implicated in key cancer functions, suggesting that they are, indeed relevant to carcinogenesis. Another group of potential cancer drivers identified here are proteins involved in the immune system. With the growing appreciation of the importance of malfunction of the immune system in allowing cancer progression[34,48,49], the immunity genes identified here can be used to develop a series of specific hypotheses of how modifications of their interaction patterns many modify immunity response to specific cancers. Analysis of all the genes with cancer driver interfaces identified in this work is ongoing in our labs, but in the meantime we provide a complete list of such genes in the S1 Table and S1 Text, as well as in our on-line resource Cancer3D[50], inviting other groups to analyze, confirm or refute our predictions.

It is important to note that the analysis presented here was limited to high quality interfaces, predicted either from solved structures or from high quality homology models. However, about 70% of the human proteome currently has no high quality structural coverage. This fraction of the proteome includes both low complexity or disordered regions, and protein regions without reliable templates to model their 3D structures. Also, structures of many complexes are still unknown. In these cases, even if we know the structures of the subunits, we cannot define the PPI interfaces and these proteins were not included in our analysis. Finally, even though we did not explore this issue here, there are other mutations that can have an impact on PPI interfaces, such as in-frame indels or silent mutations[51]. Therefore, the results presented here represent only the tip of the iceberg of what can be achieved by including structural data in the analysis of cancer mutation profiles. We expect that our method will improve not only as more cancer genomes are added to existing repositories (increasing the statistical power of the analysis), but also as the structural coverage of the human proteome increases. We expect such increase to come from both new experimentally determined structures in public databases and the use of better modeling tools[52,53].

Another important results of our analysis is that we found that tumors with mutations in the same driver gene can have surprisingly different behavior and outcomes depending on the specific PPI interface affected by the mutation. This adds to a growing body of evidence suggesting that the current gene-centric paradigm in biology, while successful in some cases, will probably not be enough to explain the complex genotype-phenotype relationships underlying a vast array of complex traits[54–59]. In the case of cancer, for example, it is known that the two most common mutations in PIK3CA, E545K and H1047L, contribute to carcinogenesis through different mechanisms[45]. The same is true for different types of mutations in KRAS[60] or, as we have shown here, for mutations in EGFR or TP53. All of the above suggests that in order to predict the outcome of a patient or the best treatment option we will need to have more detailed knowledge about the consequences of a specific mutation than just the identity of the cancer driver gene where it is located. Such increase in detail and knowledge should include, in the case of missense mutations, not only information about the protein domain or PPI interface of the gene being altered, but also data about mutations in other regions of the network, as these can also influence the phenotype of a driver gene through synthetic interactions[61].

Finally, one must keep in mind that, while this work has focused on the analysis and interpretation of missense mutations, there are many other types of variations that can act as cancer drivers and have a significant impact in the outcomes of cancer patients. Examples include promoter mutations[62], copy number variations[63], silent mutations[51] or small insertions or deletions. It is likely that different types of variations of the same gene will have different consequences and, therefore, could need different therapeutic approaches. A clear example of this phenomenon are TP53-driven tumors. As we show here, it is likely that patients with missense mutations in this gene have different outcomes depending on the specific region of TP53 that is mutated, suggesting that they might need different treatments. Nevertheless, a patient whose tumor is driven by a TP53 copy-number loss might benefit from yet another therapeutic approach that would not help any of the above[64]. Therefore, in order to identify the optimal treatment of each patient we will need to integrate and properly analyze all molecular consequences of the different types of mutations present in its tumor.

## Materials and Methods

### Code and data availability

All the supplementary information, the raw data and the algorithms used in this manuscript, as well as the results presented, can be downloaded from http://github.com/eduardporta/e-Driver. The link to the Dropbox folder containing all the raw data can be found in the README.md file. All the statistical calculations were done using R 3.1.0. All figures have been generated using the R package “ggplot2”.

### Mutation data

We downloaded level 3 mutation data from the TCGA data portal (https://tcga-data.nci.nih.gov) for 5,989 tumor samples that belong to 23 different cancer types (Table A in S1 Table). We then used the Variant Effect Predictor tool to derive the consequences of each mutation in the different protein isoforms where it mapped[65]. We used gene and protein annotations from ENSEMBL version 72. We identified a total of 868,508 missense mutations in 19,196 proteins. Note that we only analyzed the longest isoform of each gene in order to minimize problems related to multiple testing.

### Identification of the protein-protein interfaces

We identified 18,651 protein structures with multiple chains in PDB (as of May 2014). Then, we analyzed all such structures to find the residues implicated in PPI interfaces. To that end, we defined a protein-protein interface in a chain as all the residues with a heavy atom within 5 angstroms of another heavy atom from a different chain, an intermediate value between the 4 and 6 angstroms seen in other references[31,66]. If a chain was in contact with multiple other chains, we defined a different interface for each chain-chain pair. Note that any specific interface does not have to be linear in sequence and that the same residue can be involved in multiple interfaces from different structures.

The complete dataset containing all the PPI structures and models from Interactome3D was downloaded on April 30^th^ 2015. As described previously, protein-protein interfaces are defined as those residues in a chain whose atomic distance falls within 5 angstroms from the partner chain. Since Interactome3D uses Uniprot protein sequences while we use ENSEMBL, we had to map the coordinates from one to the other. In order to do that we compared the two sets of sequences and kept only those interfaces in Uniprot proteins whose sequence matched exactly a protein from ENSEMBL. While this reduced significantly the number of potential interfaces from Interactome3D, from 26,383 interfaces to 11,169, it ensured that the results obtained with each dataset would be comparable.

### Structure mapping

The mapping between ENSEMBL and PDB is the same as the one used in Cancer3D. Briefly, we queried the full PDB (March 2014), including non-human proteins, with every protein from ENSEMBL using BLAST. Every time we identified a PDB-ENSEMBL pair with an e-value below 1e^-6^ we used the BLAST output to map the residues from the ENSEMBL sequence to the PDB structure[50].

### e-Driver analysis

We used e-Driver[22] to identify interfaces that are enriched in somatic missense mutations. The algorithm calculates the statistical significance of deviation from the null hypothesis that the mutations are distributed randomly across the protein using a right-sided binomial test:

$$P\left(MR,\;MT\right)=\;\left(\begin{array}{c} MT\\ MR \end{array}\right)\;{\left(P_{MutReg}\right)}^{MR}{\left(1-P_{MutReg}\right)}^{MT-MR}$$

Where “P_MutReg_” is the ratio between the number of residues involved in the interface and the number of aminoacids in the entire protein, “M_R_” is the number of mutations in the interface and “M_T_” is the total number of mutations in the protein. Since it is possible that only a fraction of the protein is covered by the structure, we adjusted the algorithm to limit all the parameters to the structure-mapped region of the protein (for example “M_T_” refers to the total number of mutations in the region of the protein covered by the specific structure being analyzed, not the absolute total of mutations in the protein). The final step consists in correcting all the p values for multiple testing using the Benjamini-Hochberg algorithm. We considered as positives all of the interfaces with a q value below 0.01.

### PPI Network analysis

Given the large number of available human PPI networks and the variability in their quality, we decided to use 16 different networks from 7 different sources (Figs H-K in S1 Text): HPRD[67], Biogrid[68], STRING[69], HumNet[70], PSICQUIC[71], one PPI derived from unbiased experiments as well as curated literature[43] and another network derived from *in silico* predictions of PPI based on structures[72] (which we will call “Kotlyar” from this moment). Three networks, STRING, HumNet and Kotlyar have scores that approximately correlate with the probability of the interaction being true. Therefore, we decided to divide these networks in different subsets, by selecting only those interactions above a certain threshold (Figs H-J in S1 Text). We then calculated the different protein properties (node degree and node and edge betweenness) in each network using the R package “iGraph”.

We identified several potentially confounding factors that could explain differences in network properties of the different proteins. For example, there is a clear bias introduced by the fact that we can only analyze proteins with structurally covered regions (either by direct experimental structures or homology modeling). Another potential confounding variable is the number of publications of each protein, as it seems to correlate with the number of interactions[43]. In order to do that, we used the e-tools from Pubmed to retrieve the number of papers mentioning each gene symbol in the title or the abstract. We also took into account whether the gene is a known cancer driver or not, as cancer driver genes are also known to have high degree and betweenness centrality[73]. Finally, we fitted the aforementioned variables, as well as whether the gene was an interface driver or not, into a generalized additive model (using the R package “gam”) and calculated the correlation of each variable with the degree or betweenness centrality in every network.

### Distance between known and novel potential cancer driver genes

We measured the distance between known cancer driver genes and genes identified by e-Driver not yet known to play a role in carcinogenesis in the different networks. To that end we calculated the distance between both groups of genes using the random walk with restart (RWR) algorithm. The random walk on graphs is defined as an iterative walker’s transition from its current node to a randomly selected neighbor starting at a given source node. This algorithm has been extensively used for the predictions of disease-associated genes[74] as well as the analysis of cancer genomes[75–77]. It also allows the restart of the walk from the source nodes at each time with probability “r”. For a detailed explanation of the effects of different “r” values see Fig L and Fig M in S1 Text. The random walk is described by the equation:

$$p_{t+1}\;=\;(1-r)*W*p_{t}+r*p_{0}$$

Where W is a column-normalized adjacency matrix of the graph, p_t_ is a vector in which the i-th element holds the probability of being at node i at time t and p_0_ is the initial probability vector. This vector has value 0 if the gene is not a known driver, and value 1/D if the gene is a known driver, where D is the number of known driver genes in the network. The algorithm iterates the equation until the L_1_ norm between p_t_ and p_t+1_ is less than 10^−6^. Then, we added the probabilities of all the candidate driver genes identified by e-Driver and compared it to 10,000 groups with the same number of random genes to calculate empirical right-sided p-values.

### Protein expression and clinical data analysis

We downloaded level 3 clinical and protein expression data, whenever it was available, from the TCGA data portal. Then, for each statistically significant interface, we classified each sample into one of three groups: samples with mutations in the interface, samples with mutations in other regions of the same protein, and samples with no mutations in that protein.

Finally, in the case of proteomics data, we used a two-sided Wilcoxon test to identify proteins with statistically significant differences between the first group and the other two. As for the clinical data, we used the Cox proportional hazards model from the R package “survival” to estimate whether mutation of a specific interface was a predictive feature for survival (p < 0.01) after correcting by age.

## Supporting Information

S1 Text Supplementary Text with additional details and Figures about the methods, the analysis and the results. (DOCX)

S1 Table Supplementary Table with descriptions of the datasets, all the results generated by e-Driver, statistics about the overlap with Interactome3D and survival data analysis. (XLSX)
