# Supplementary material for: A Pan-Cancer Catalogue of Cancer Driver Protein Interaction Interfaces
Source: PLoS Comput Biol. 2015 Oct 20;11(10):e1004518. doi: 10.1371/journal.pcbi.1004518 (PMC4616621; doi:10.1371/journal.pcbi.1004518)
Supplement: S1 Text — (DOCX) [file pcbi.1004518.s001.docx]

Supplementary Text for the manuscript

**A Pan-cancer catalogue of driver protein interaction interfaces**

Eduard Porta-Pardo^1*^, Luz Garcia-Alonso^2*^, Thomas Hrabe^1^ Joaquin Dopazo^3,4,5+^ and Adam Godzik^1+^

^1^Bioinformatics and Systems Biology Program, Sanford-Burnham Medical Research Institute, 10901 North Torrey Pines Road, La Jolla, CA, 92037, USA

^2^European Bioinformatics Institute (EMBL-EBI), Wellcome Trust Genome Campus, Cambridge CB10 1SD, UK

^3^ Computational Genomics Department, Centro de Investigación Príncipe Felipe (CIPF), Valencia, Spain.

^4^ Functional Genomics Node, (INB) at CIPF, Valencia, Spain.

^5^ Bioinformatics of Rare Diseases (BIER), CIBER de Enfermedades Raras (CIBERER),

Valencia, Spain

*These authors contributed equally to this work

^+^To whom correspondence should be addressed: jdopazo@cipf.es / adam@godziklab.org

**1 - The Pan-cancer dataset**

We compiled the mutation profiles of 5,989 cancer patients from 23 different projects of The Cancer Genome Atlas^1^. Not all of the projects have the same number of samples (Fig A), with breast adenocarcinoma being the most represented cancer type and having almost twice as many samples as the next cancer project, lung adenocarcinoma.


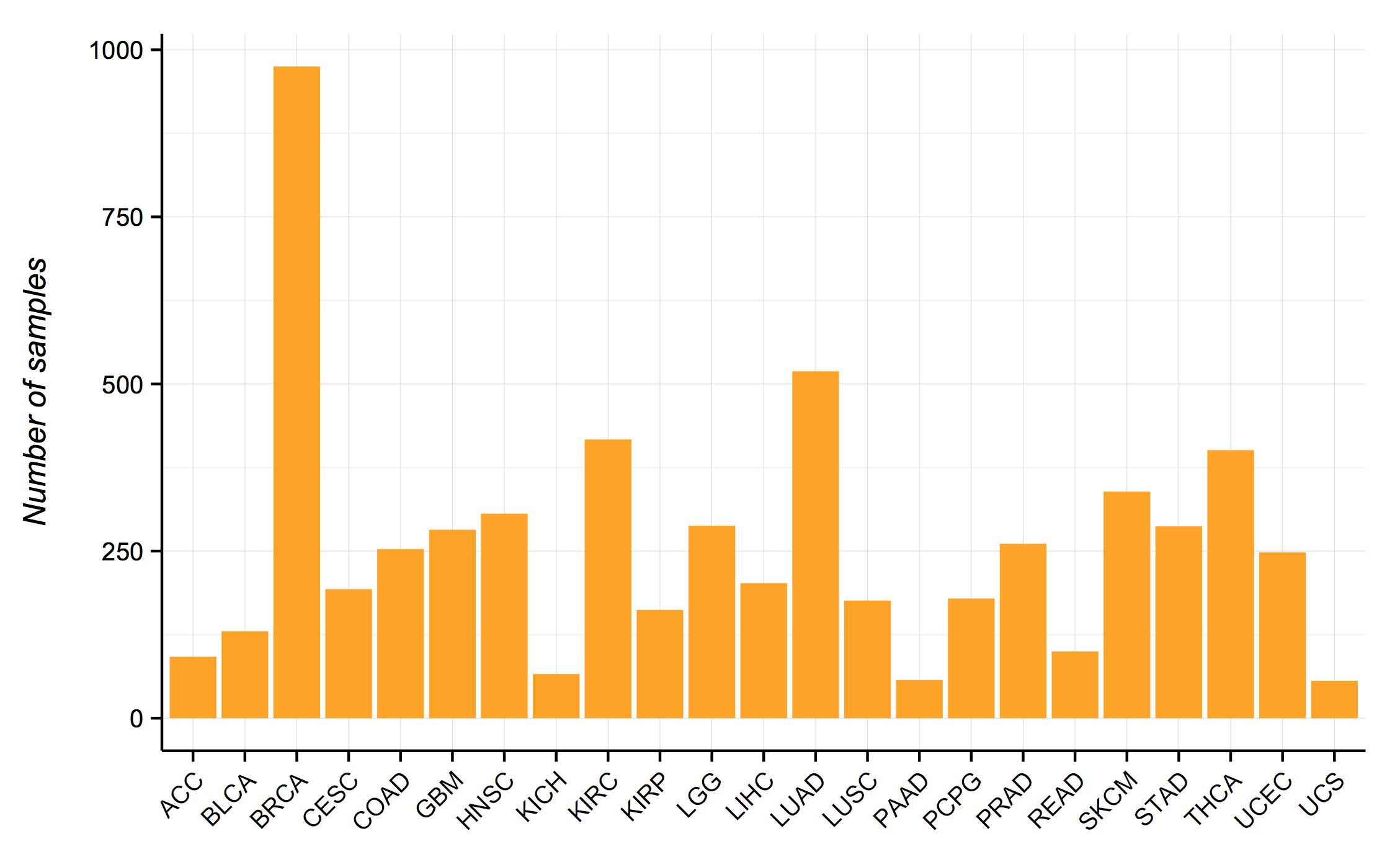


Figure A– Number of tumor samples analyzed from each cancer type.

We also observed, in agreement with other reports^2^, that the number of mutations per sample is highly heterogeneous between both samples of the same cancer type as well as between different cancer projects (Fig B). Nevertheless, some cancer types, such as lung adenocarcinoma or melanoma, had consistently high number of mutations.


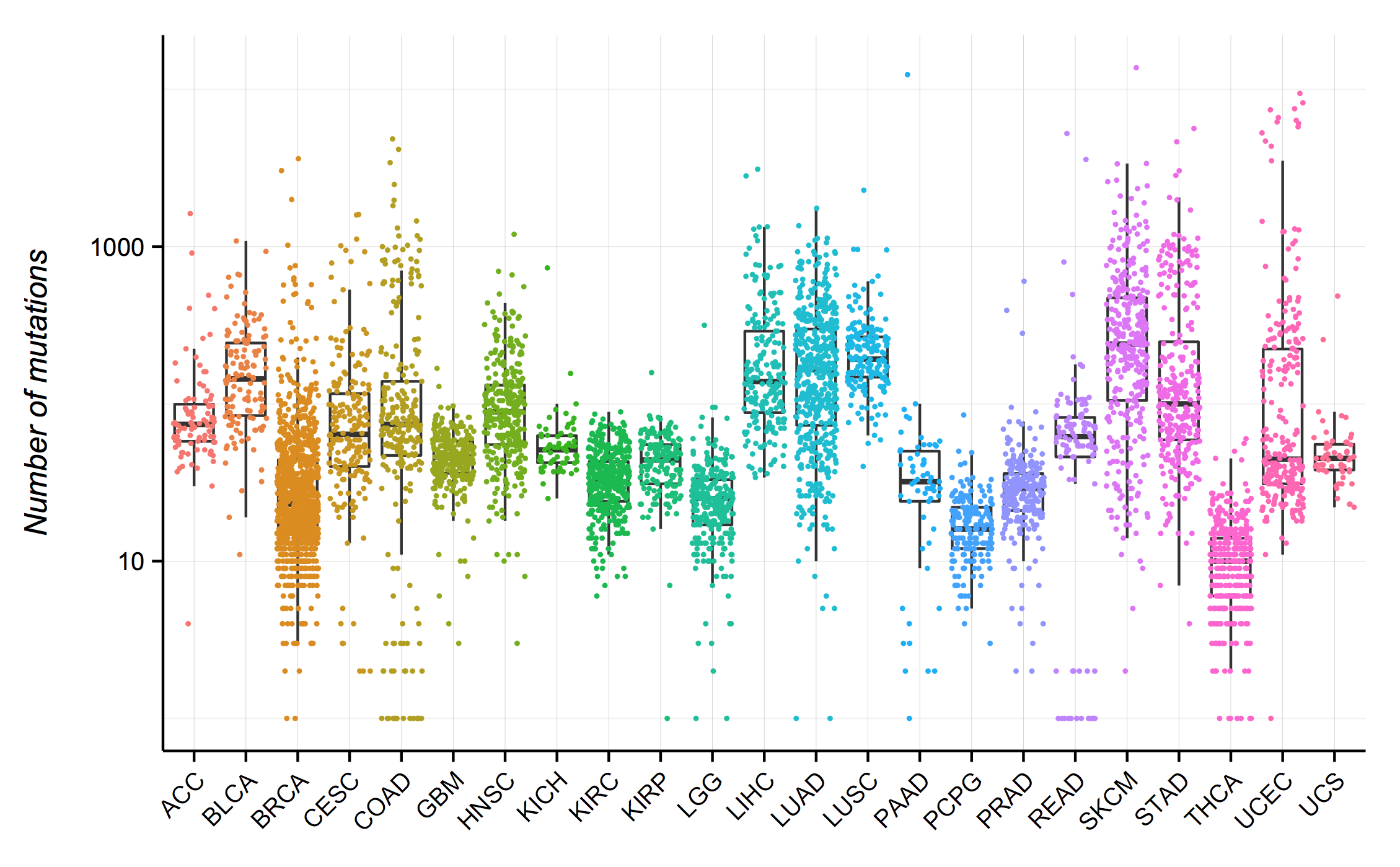


Figure B - Number of mutations in each sample. The y axis shows the number of mutations (logarithmic scale) and the x axis the different cancer projects. In each case the boxplot shows the median number of mutations as well as one standard deviation in each direction. Each dot represents an individual tumor sample and is colored according to the cancer project where it belongs.

**2 - Structural coverage of the human proteome**

Our analysis is strongly dependent of the coverage and quality of structural annotations in the human proteome. The whole process to obtain this coverage is explained in the Online Methods, but briefly, we used BLAST^3^ to query all the protein sequences from PDB^4^ (human or not) with each of the human protein sequences from ENSEMBL v72^5^. Only alignments with an e-value below 1e-6 were considered for further analysis, and their output was used to map the residues from ENSEMBL to their corresponding positions in three-dimensional structures.

Using this pipeline, we covered approximately 30% of the human proteome (Fig C, last bar), but the coverage dropped to only 26% for high-confidence driver (HCD) genes^6^. The lower coverage of HCD genes is probably caused by several different factors, but one of them is likely the higher disorder levels of these proteins^7^, which makes them more difficult to crystallize. We found a slight enrichment of mutations, between 5% and 10%, in the structurally covered fraction of the proteome (Fig D). Remarkably, this enrichment was more pronounced, between 50% and 200%, in structurally solved regions of HCD genes, which probably reflects significance research focus on these genes.

We then looked for protein-protein interaction (PPI) interfaces using the three-dimensional coordinates from PDB. We defined a PPI interface as all the aminoacids of a chain (A) in a PDB structure that have a heavy atom from a different chain (B) less than 5 angstroms away from a heavy atom of an aminoacid. The 5 angstrom threshold was selected as the average value of similar publications^8^. It is important to note that this approach has certain limitations and sources of potential false positives. First, we are using BLAST to find structures from homologous proteins and maximize our coverage of the proteome. However, it is possible that two proteins with homologous domains use different interfaces for their interactions. Another issue comes from the fact that we are using all the chains in a PDB file to define interfaces. There are many structures in PDB that have dimers of the same protein interacting between them that do not represent biologically relevant interactions, but are crystallographic artifacts. In these cases we would define a PPI interface that might not be biologically relevant (though that is still an issue open for discussion^9,10^). That being said, a similar pipeline has been used to predict novel interactions that have been later experimentally verified^11^. Moreover, the fact that the regions identified in our analysis have, apparently, a non-random accumulation of somatic mutations argues in favor of these regions having a defined biological function, likely mediating a PPI interaction.

This second part of our pipeline yielded over 120,000 PPI interface aminoacids in more than 10,000 different proteins. These interfaces represent approximately 6% of the proteome, and have roughly the same proportion of cancer somatic mutations, though, again, there is a slight enrichment in mutations in these regions just as there is for whole structures. When looking at HCD genes, we found that 7% of them are part of a PPI interface, though they have a disproportionate number of mutations, with enrichments ranging from two to over three-fold depending on the cancer project. This confirms that disruption of PPI interfaces is a major carcinogenic mechanism of mutations in HCD genes.


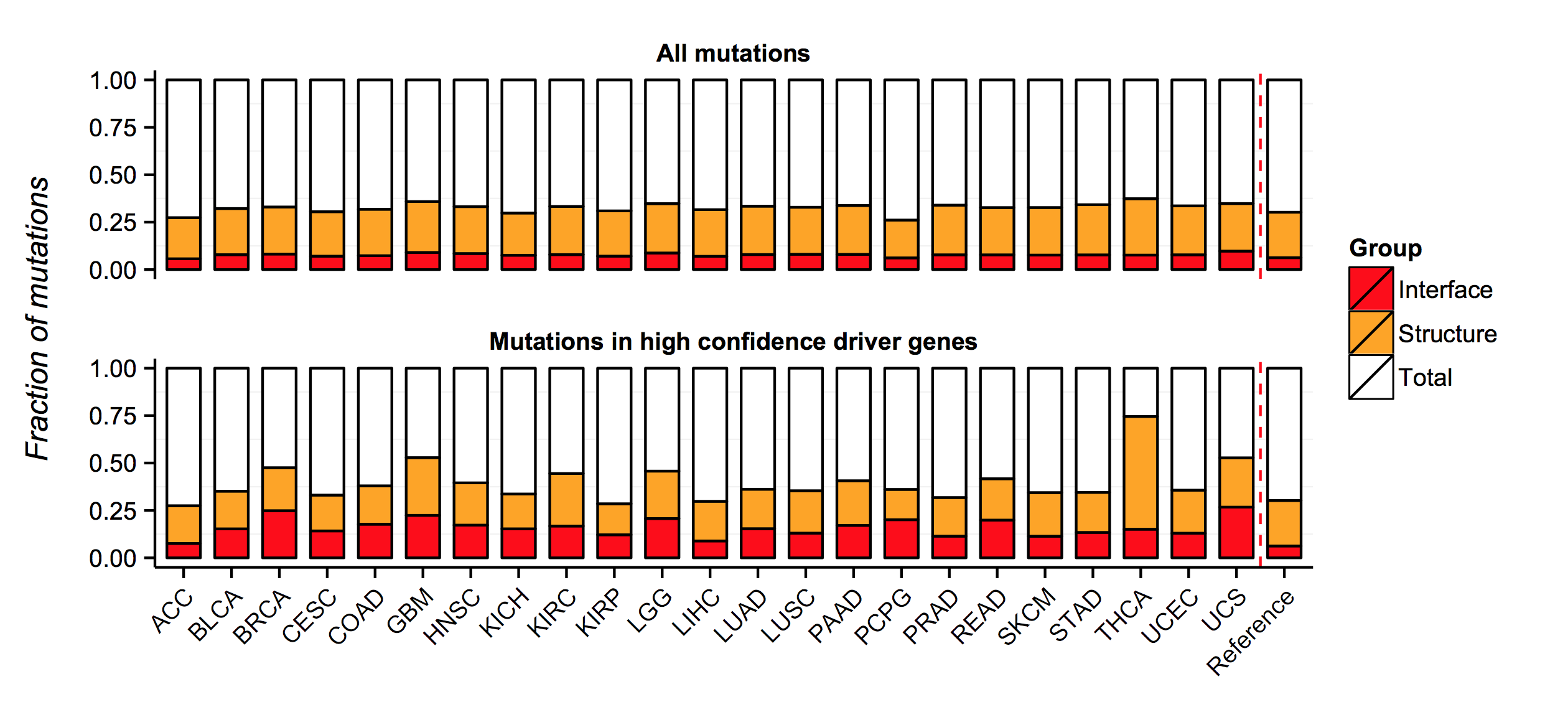


Figure C – Distribution of mutations across the proteome in terms of structural coverage. The y-axis shows the fraction of mutations mapped to the different regions of the proteome, whereas the x-axis shows the different cancer projects. The overall coverage in the proteome is shown in the last histogram, separated by a red dashed line and marked as “Reference”. The top histogram shows the distribution for all the mutations in each project, while the bottom histogram shows the distribution for high confidence driver genes only.


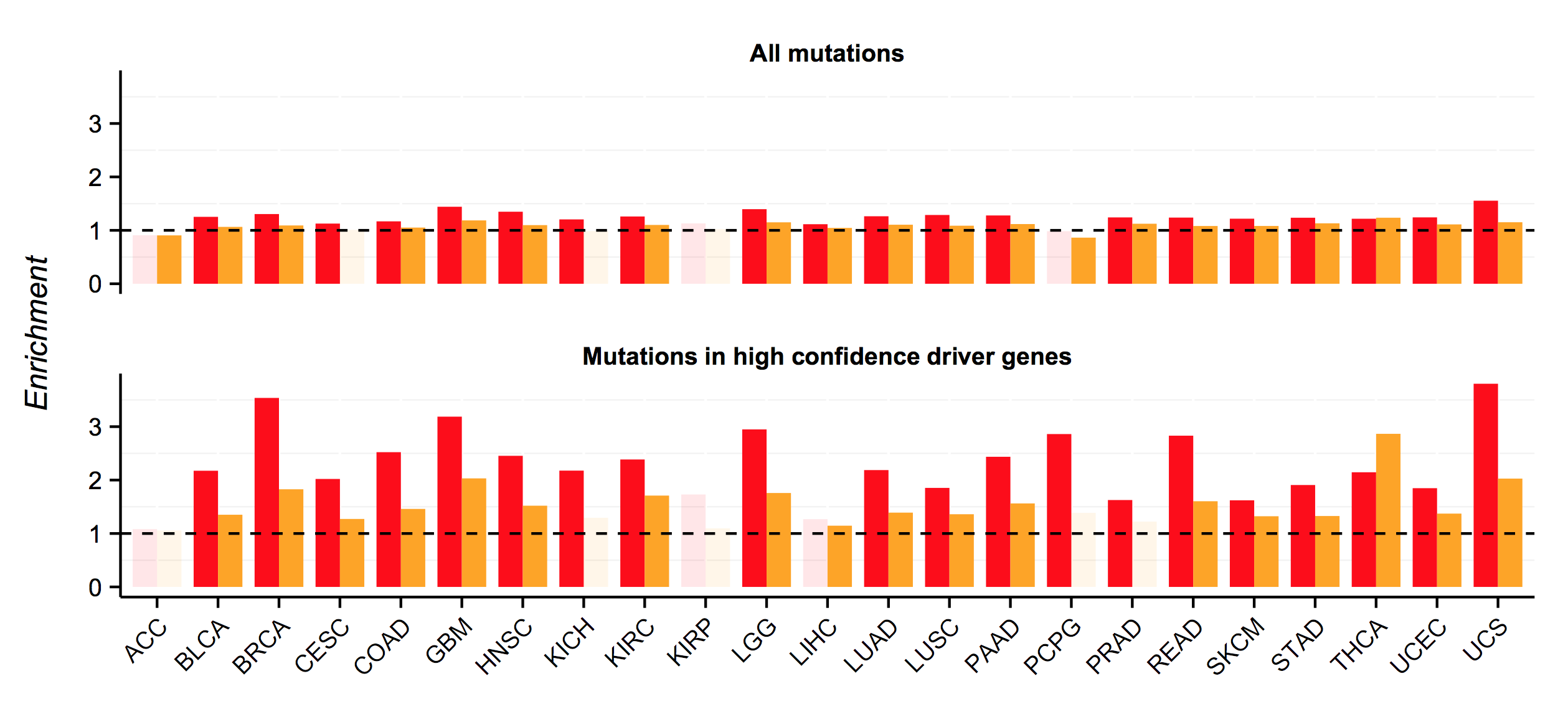


**Figure D – Structurally solved regions from known driver genes are enriched in missense mutations**. This histogram shows the ratio between observed and expected mutations in structurally solved regions (orange) and PPI interfaces (red) in the different cancer projects. At the top one can see the relative enrichment when taking into account all missense mutations and at the bottom when using only known cancer driver genes. Bars are transparent for analyses where there are no statistically significant (p < 0.01, Binomial right-sided test) differences between observed and expected mutations.

**3 – Analysis with other datasets of structurally characterized interfaces**

In order to validate our method and findings we repeated the analysis using a different dataset of structurally characterized interfaces than that used in Cancer3D and described above. To that end, we downloaded the data from Interactome3D^12^ and extracted the interfaces.

After mapping the proteins to ENSEMBL we compared the properties of both interface datasets. As shown in Fig E, the number of both, proteins and interfaces, is much higher in Cancer3D (the dataset obtained with our own pipeline and described above) than in Interactome3D. This is probably because Interactome3D only includes interfaces with both partners in a known interaction map to the same structure. In the case of Cancer3D we are less stringent and we consider the interface even when no known interaction partner of a protein maps to the same PDB structure. For example, if proteins A and B interact, Interactome3D will count an interface in protein A only if there is a structure where it interacts with protein B. On the other hand, in Cancer3D there we include interface for protein A as long as it can be mapped to a structure where an interaction is described, regardless of whether protein B can also be mapped to that structure.


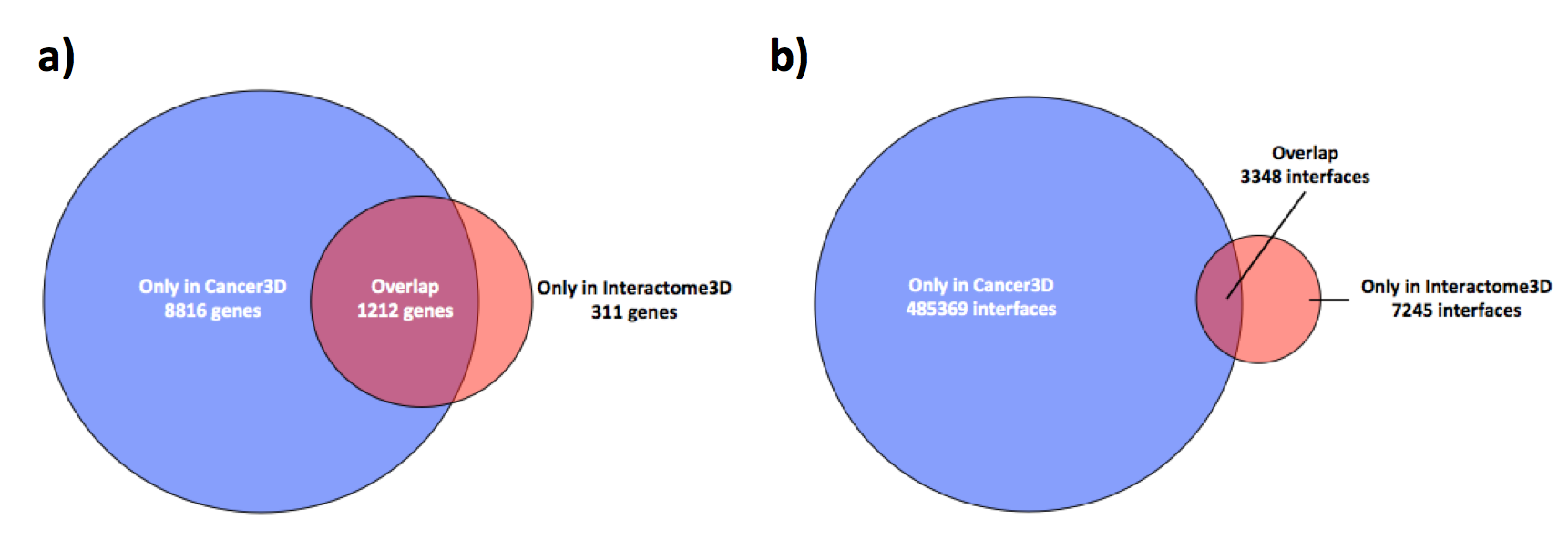


Figure E - Overlap between Interactome3D and Cancer3D. a) Venn diagram showing the overlap in terms of genes between Cancer3D and Interactome3D. Cancer3D has 8816 unique genes, whereas Interactome3D has 311 unique genes and the overlap between the two is 1212 genes. b) Venn diagram showing the overlap in terms of interfaces between datasets. Cancer3D has 485369 unique interfaces, while Interactome3D has 7245 unique interfaces and the overlap is 3348. Most unique interfaces in Interactome3D are due to the fact that it used a more recent version of PDB with more structures.

We then looked for differences between the structural mappings of both datasets (i.e. if there is a region of a protein that can be mapped to a structure, where exactly does each dataset consider that the structure is located within the protein). In order to do that we measured the overlap between the start and end positions of each protein – PDB mapping in Cancer3D and in Interactome3D (Fig F). We found that both datasets were very consistent when mapping structures to proteins, as more than 97% of the mappings had over 90% overlap. Interestingly, the exact positions of the interfaces where slightly different between the two datasets, with most interfaces having a level of agreement ranging from 50% to 95%. This is probably because Interactome3D builds binary models for PPIs considering the protein sequences, whereas in Cancer3D we use the direct output from BLAST to map the interfaces between homologous proteins.


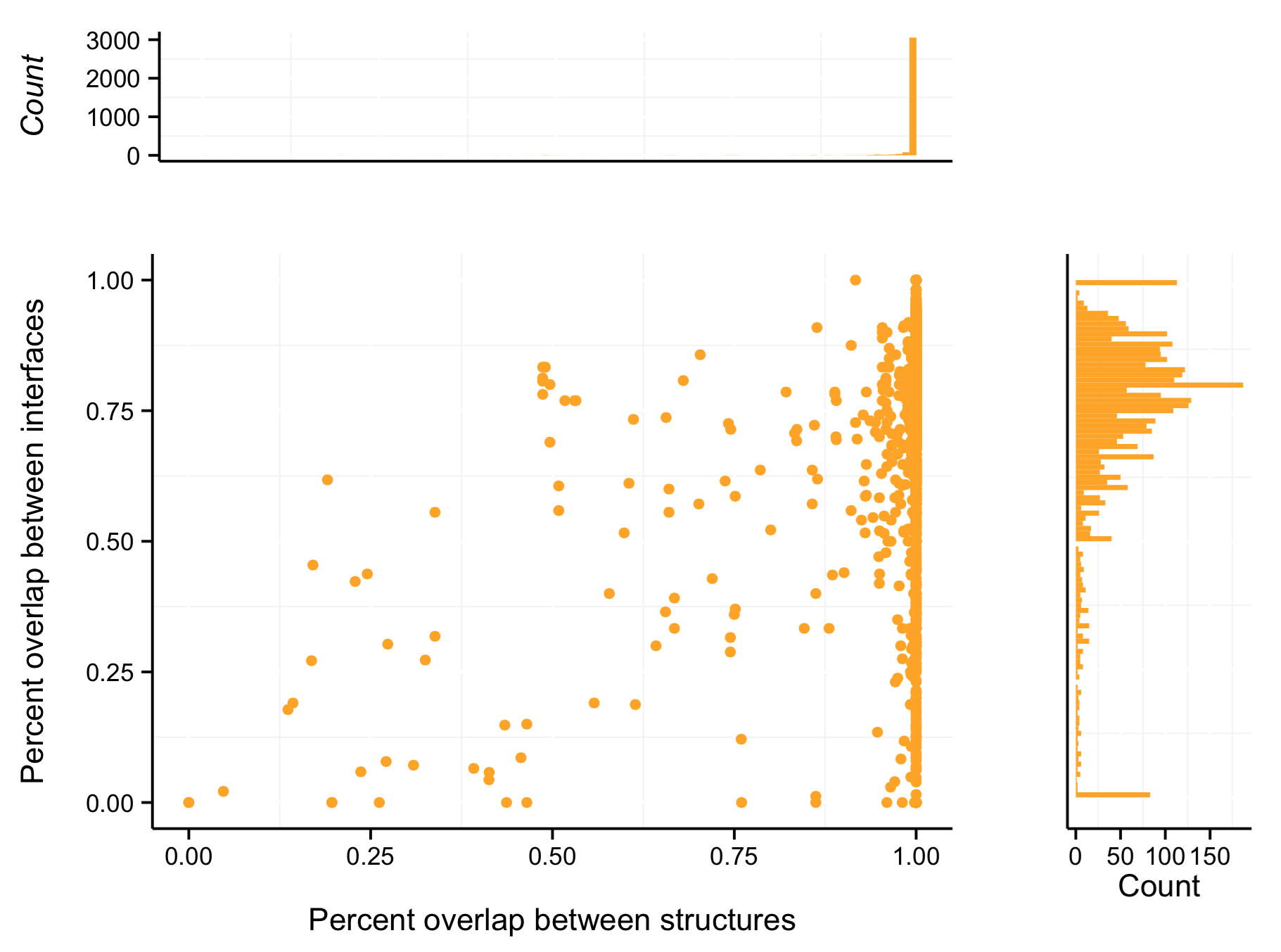


Figure F - Comparison of structural mappings from Interactome3D and Cancer3D. Both datasets have a high level of agreement about where each structure should be located within a protein (X axis and top histogram), but there are some differences between them regarding the exact positions of the interface residues (Y axis, right histogram).

We then reviewed the list of “interface driver” genes identified using data from Interatome3D. As shown in Fig G, most genes with significant enrichment of mutations in interfaces defined by Interactome3D were also identified when using data from Cancer3D. Out of 19 genes identified when using data from Interactome3D, 8 were also found when using data from Cancer3D. The remaining 11 were either very close to the significant threshold (p < 0.05) in the Cancer3D analysis (4 genes), or were not included in the analysis because the structures were added after the analysis was completed (6 genes). The only interface that was not identified when using data from Cancer3D belongs to TP63 when mapped to the PDB file 3Q01, a structure showing a dimer of TP53 molecules. The Interactome3D pipeline assigns the interface to the TP63 residues between 365 and 386. On the other hand, this same region is not mapped to the structure in Cancer3D, as it is located in a gap of the BLAST alignment, hence the difference between the two analyses.

Overall, even though we identified some differences between the results obtained when analyzing interfaces from Cancer3D and Interactome3D, these differences were minimal and caused by changes in how the protein sequences where mapped to structures. While this highlights the importance of curating and further developing methods to determine three-dimensional protein structures, we believe that it also shows the robustness of our approach and the e-Driver analysis.


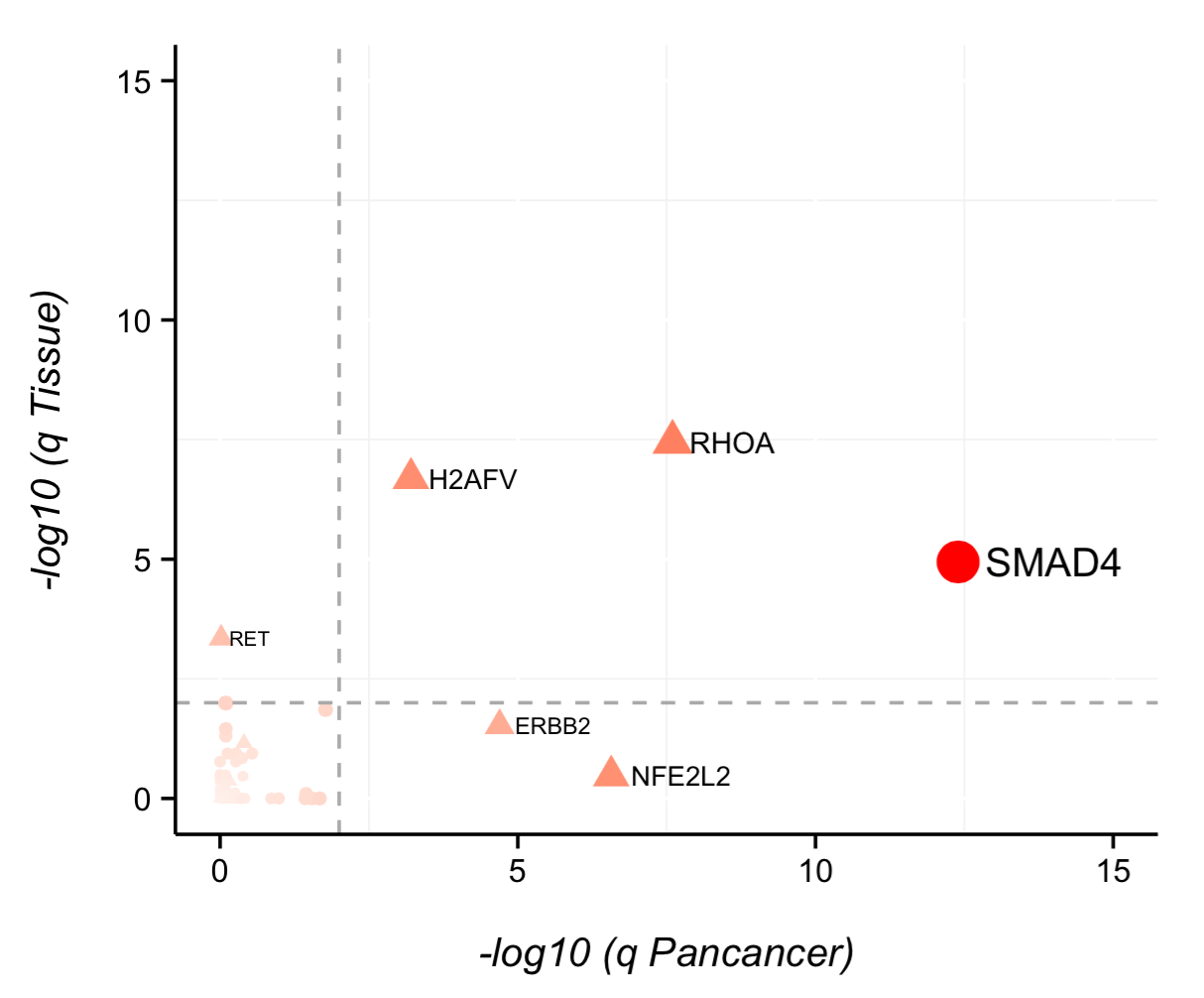


Figure G - Significant genes identified by e-Driver using interfaces from Interactome3D. X,Y coordinates reflect the q value (FDR) in the Pancancer analysis and their lowest q value in all 23 project-specific analysis, respectively, when using interfaces from Interactome3D. Gray dashed lines are located at 0.01 FDR for reference. Dots are colored, sized and labeled according to their FDR: genes with an FDR of 1 are colored in white, are smaller and have no label whereas genes with lower FDRs are more red, bigger and labeled. Note that there are *EGFR* and *ERBB2* that are not shown because their FDR value was too small (FDR < 1e-15). Genes shown as triangles were also identified when using interfaces from Cancer3D, whereas those shown as circles were not.

**4 – Network properties of interface driver genes**

Once we found that PPI interfaces are recurrently mutated in cancer driver genes, we wondered whether these interface driver genes would also have distinct features in terms of network topology. It is known that cancer driver genes, as a group, are usually PPI network hubs and occupy bottleneck positions in the interactome. To that end, we measured the degree and betweenness of the interface driver genes in 7 different biological networks: HPRD^13^, Biogrid^14^, STRING^15^, HumNet^16^, PSICQUIC^17^, one PPI derived from unbiased experiments as well as curated literature^18^ (which we will refer to as “Vidal” from this moment) and another network derived from *in silico* predictions of PPI based on structures^19^ (which we will call “Kotlyar” from this moment). Three networks, STRING, HumNet and Kotlyar have scores that approximately correlate with the probability of the interaction being true. Therefore, we decided to divide these networks in different subsets, by selecting only those interactions above a certain threshold. The thresholds for each network as well as the distribution of the scores are shown in Fig H-J.


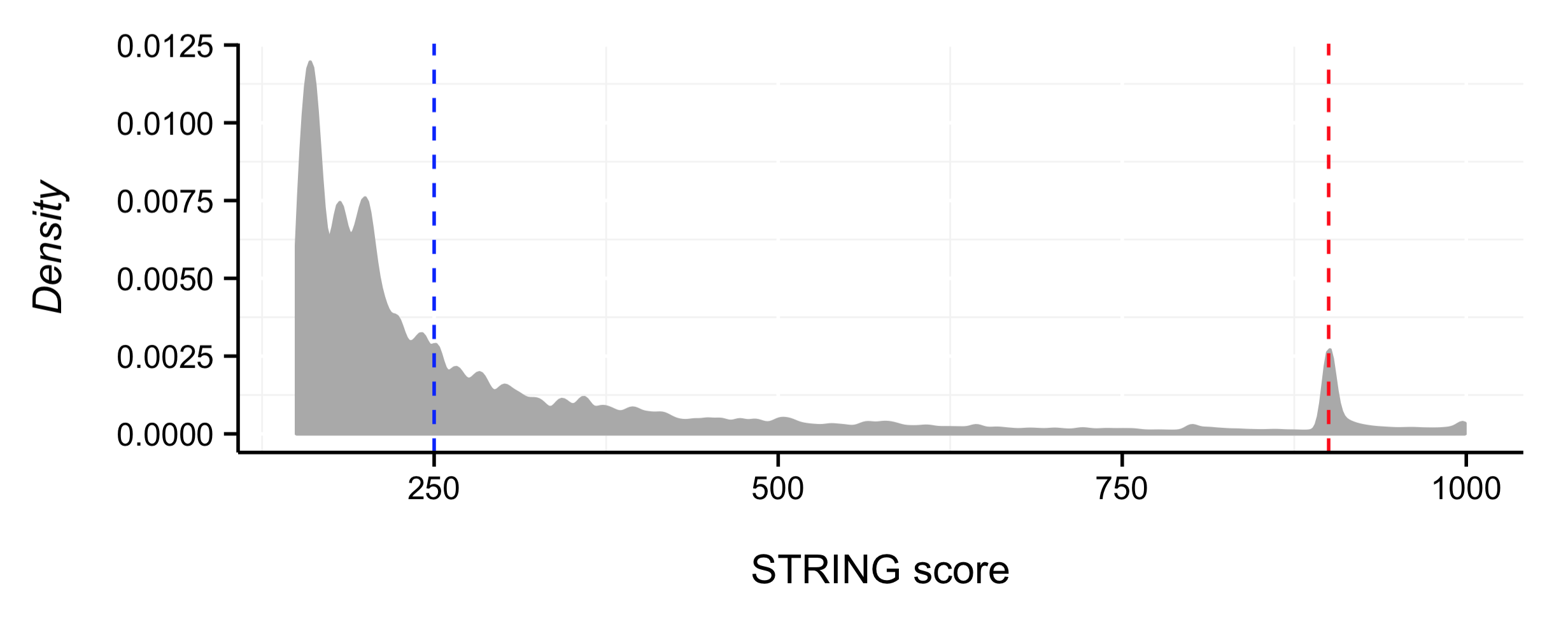

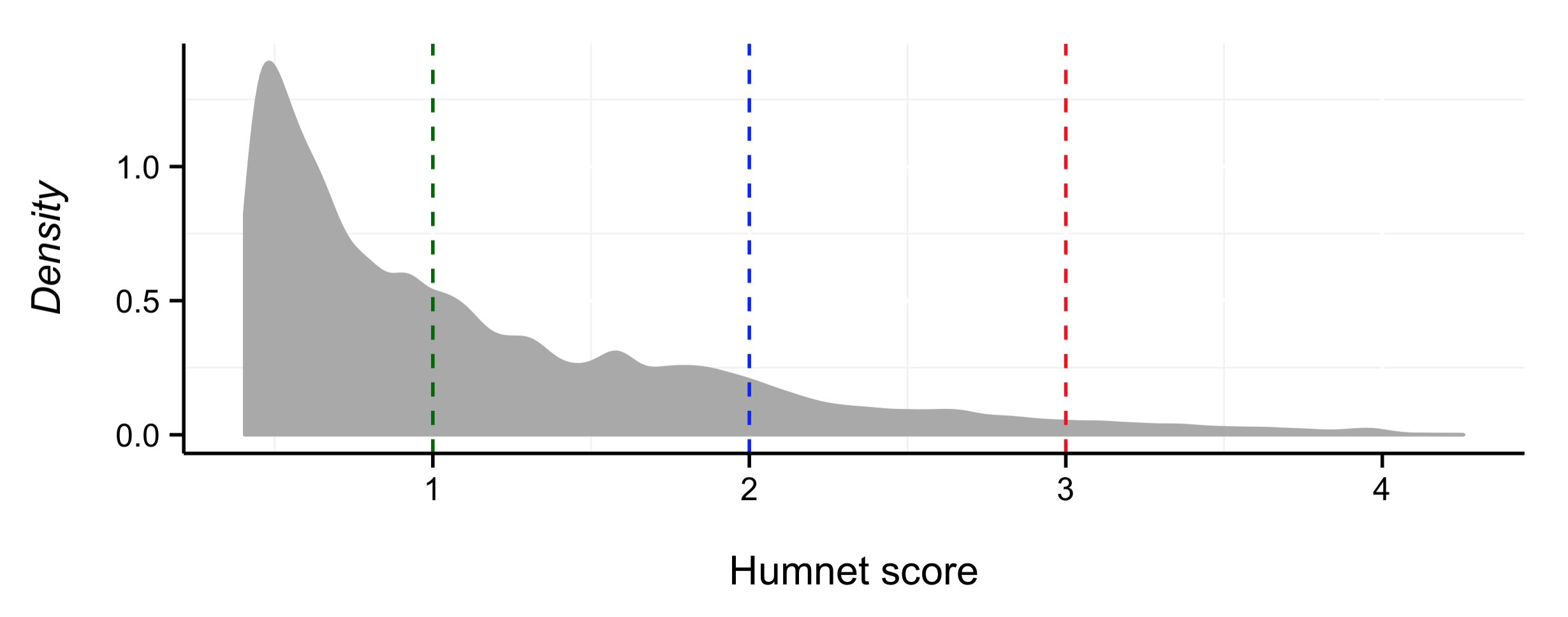

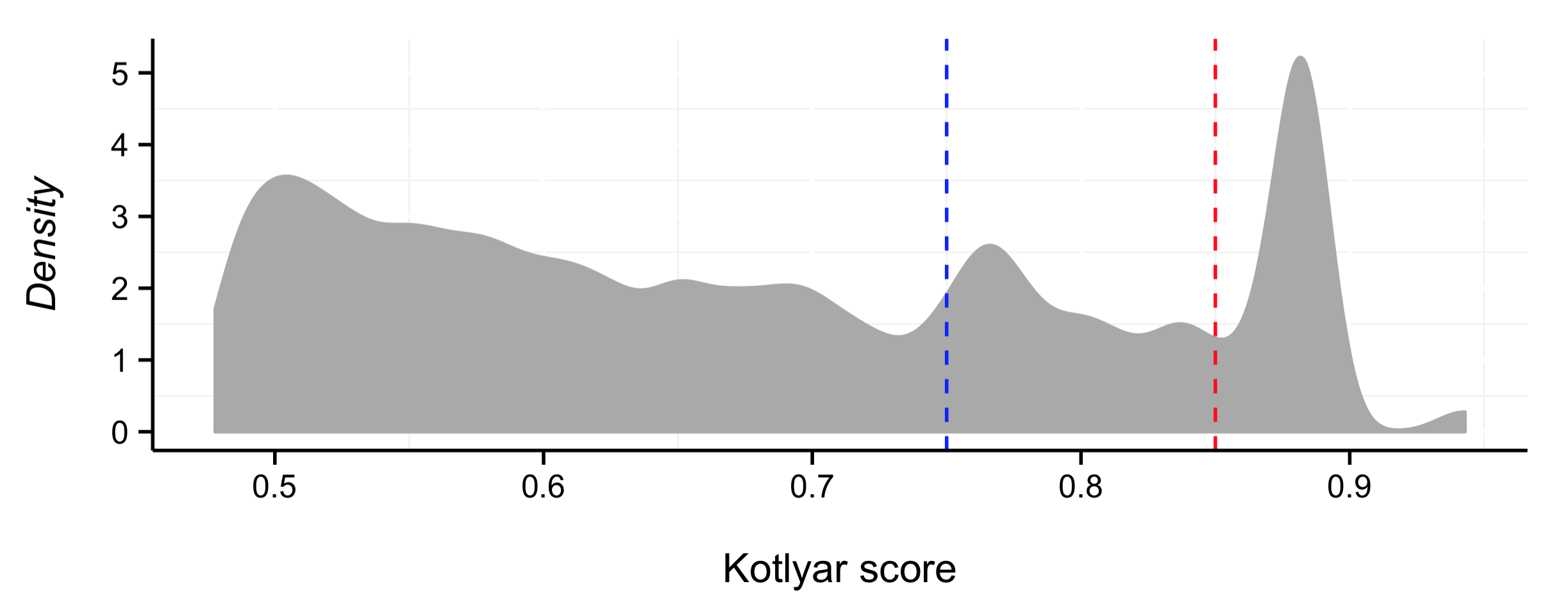


Figure H - Edge score distribution for the STRING network. We created 3 different networks from the original STRING network: 1 – STRING_0, which contains all the edges in STRING; 2 – STRING_250, which contains all the edges with score > 250 (threshold shown as vertical blue dashed line); 3 – STRING_900, which contains all the edges with score > 900 (threshold shown as vertical red dashed line).

Figure I - Score distribution for the Humnet network. We created 4 different networks from the original Humnet network: 1 – Humnet_0, which contains all the edges; 2 – Humnet_1, which contains all the edges with score > 1 (threshold shown as vertical green dashed line); 3 – Humnet_2, which contains all the edges with score > 2 (threshold shown as vertical blue dashed line); 4 – Humnet_3, which contains all the edges with score > 3 (threshold shown as vertical red dashed line)

Figure J - Score distribution for the Kotlyar network. We created 3 different networks from the original Kotlyar network: 1 – Kotlyar_0, which contains all the edges; 2 – Kotlyar_0.75, which contains all the edges with score > 0.75 (threshold shown as vertical blue dashed line); 3 – Kotlyar_0.85, which contains all the edges with score > 0.85 (threshold shown as vertical red dashed line)

**
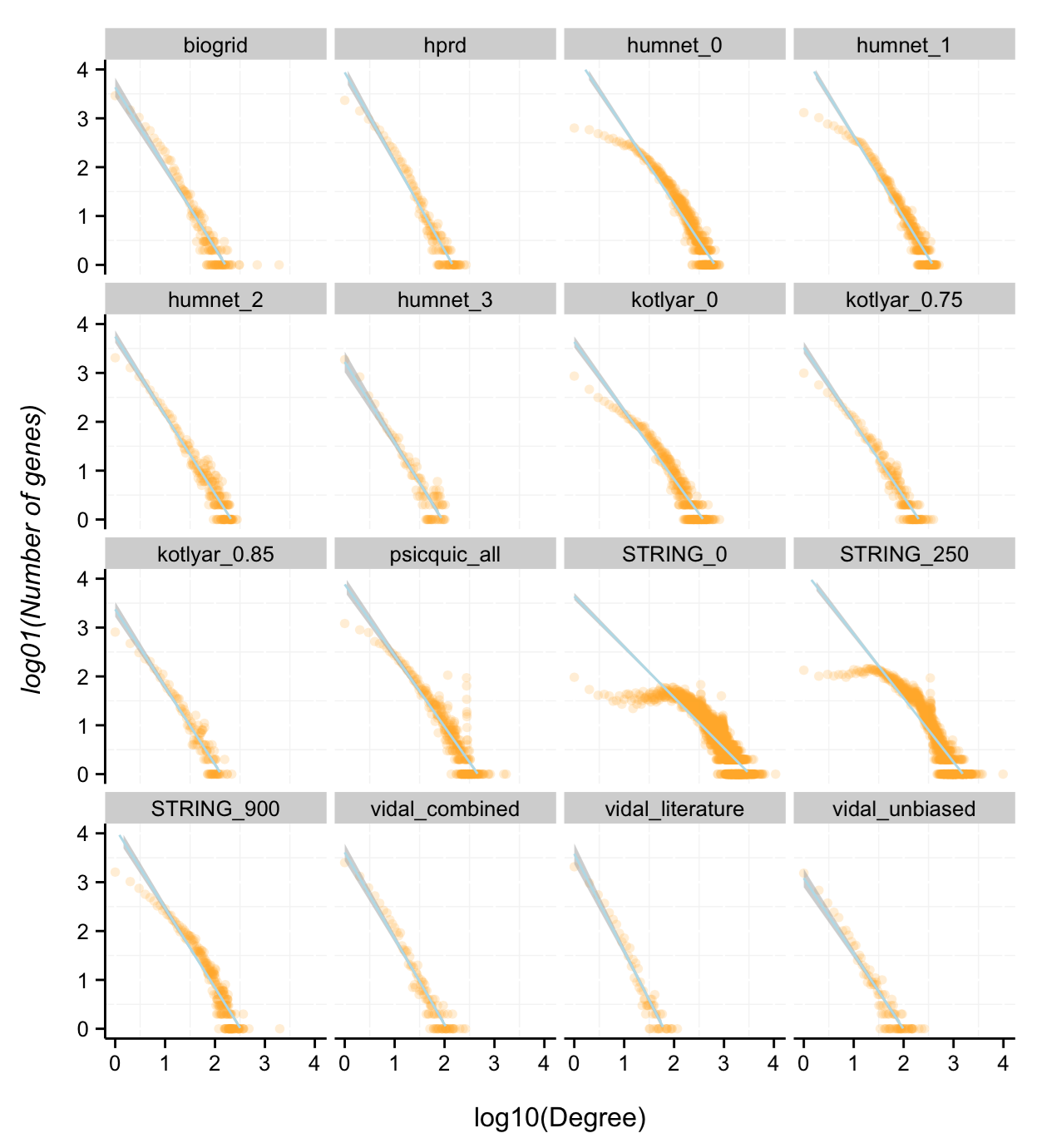
**

**Figure K – Degree distribution of the different networks**. The number of interactions per gene in each network follows a power-law distribution, with the exception of STRING_0 and STRING_250, which show a different pattern, probably because of the inclusion of many false-positives that artificially increase the number of interactions of many genes. One we take into account only interactions with very high scores (STRING score > 900), the network gets closer to a scale-free network.

We also created 3 networks from the original Vidal network: one containing all the edges (vidal_combined), another containing only the experimental edges (vidal_unbiased) and a third one with only the literature-derived edges (vidal_literature).

We then determined whether the networks are scale-free, a property known to be shared by many biological networks. This means that most genes have few interactions and a few genes have the most interactions. As shown in Fig K, most networks in our analysis do have this property, with the exception of those derived from STRING. However, note that the STRING network with the highest scores (STRING_900) comes close to being a scale-free network.

Finally, we measured the distance between known cancer driver genes and genes identified by e-Driver not yet known to play a role in carcinogenesis in the different networks. Our hypothesis here was that if the candidate driver genes are indeed contributing to carcinogenesis they would be close to known cancer driver genes, following the “guilty by association” principle, which has been used numerous times to identify disease-associated genes^20-23^.

To that end we calculated the distance between both groups of genes using the random walk with restart (RWR) algorithm. The random walk on graphs is defined as an iterative walker’s transition from its current node to a randomly selected neighbor starting at a given source node. This algorithm has been extensively used for the predictions of disease-associated genes^24^ as well as the analysis of cancer genomes^25-27^. It also allows the restart of the walk from the source nodes at each time with probability “r”. The random walk is described by the equation:

$$p_{t+1}=\left( 1-r \right)*W*p_{t}+r*p_{0}$$

Where W is a column-normalized adjacency matrix of the graph, p_t_ is a vector in which the i-th element holds the probability of being at node i at time t and p_0_ is the initial probability vector. This vector has value 0 if the gene is not a known driver, and value 1/D if the gene is a known driver, where D is the number of known driver genes in the network. The algorithm iterates the equation until the L_1_ norm between p_t_ and p_t+1_ is less than 10^-6^. Then, we added the probabilities of all the candidate driver genes identified by e-Driver and compared it to 10,000 groups with the same number of random genes to calculate empirical p-values. As shown in Fig L and Fig M, in most cases the group of candidate driver genes was closer to known driver genes than expected (p < 0.05). This result was independent of the restart probability, and supports the hypothesis that the genes identified by e-Driver can play important roles in carcinogenesis. Note that we excluded known cancer driver genes also identified by our method (such as TP53, EGFR, KRAS or ERBB2) from the set of candidate driver genes


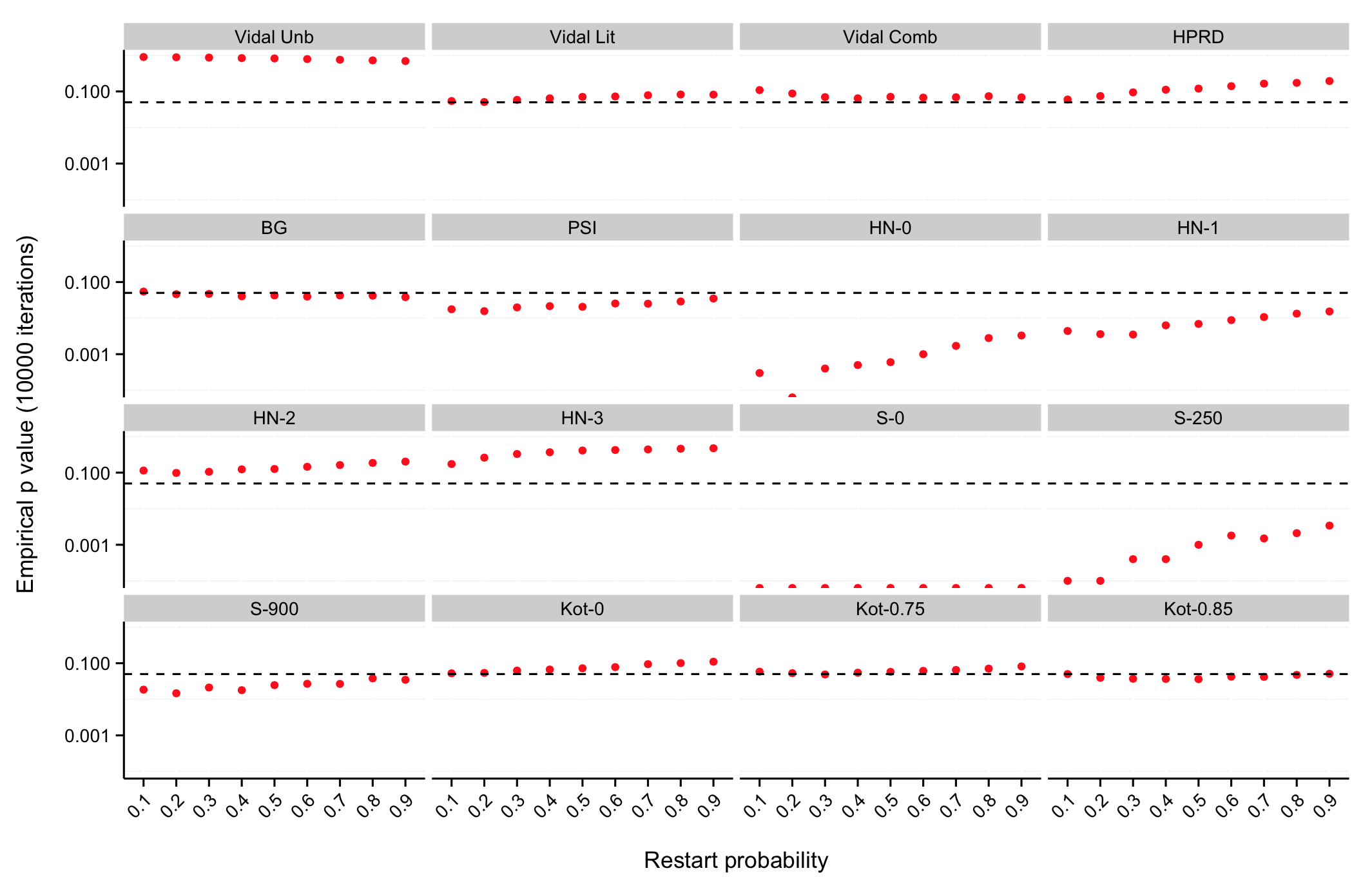


Figure L - Empirical p values obtained in the different networks depending on the restart probability. Candidate driver genes identified by our analysis are closer to known driver genes in most networks than random genes. The dashed horizontal line is located at p = 0.05 for reference. Network abbreviations are as follows: BG – Biogrid / PSI – PSICQUIC / HN – HumNet / S – STRING / Kot - Kotlyar


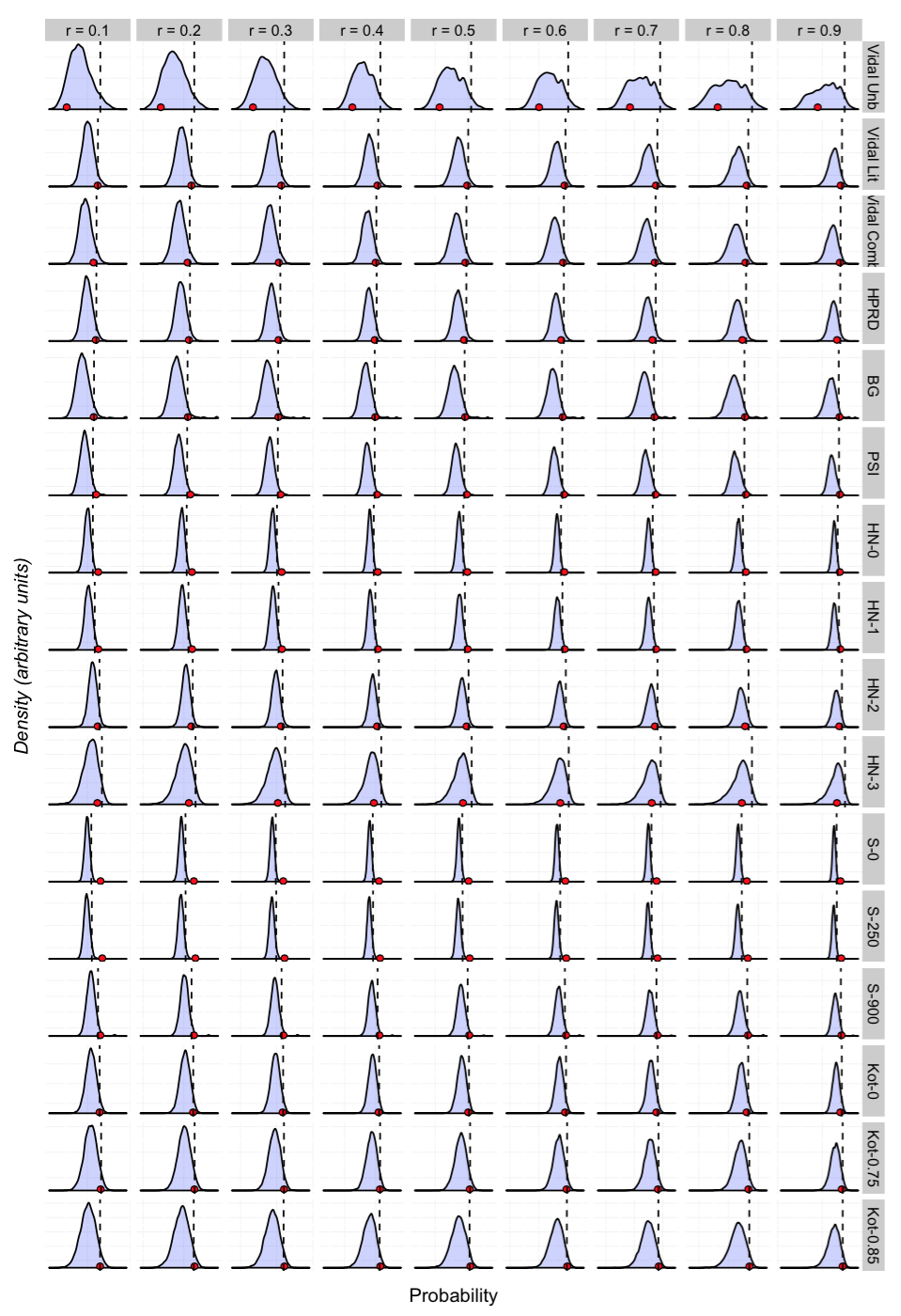


Figure M - Distance distribution for the different networks and restart probabilities. The x axis shows the probability of the random walk to end in a gene that belongs to the analyzed group (either random genes or candidate drivers) in each network (rows) depending on the restart probability (columns). Note that higher probabilities (right positions of the x-axis) correspond to genes closer to known driver genes in the network. The red dots show the probability for the group of candidate driver genes and the blue curve show the distribution for groups of random genes. The vertical dashed line corresponds to the 0.95 quantile (p <0.05 of being closer to known driver genes).

**5 – Consequences of mutations in interface driver genes**

One of the advantages of our analysis over other methods to identify cancer driver genes is that our results immediately suggest the molecular mechanism of the mutation to cause cancer: the disruption of a specific interface. This can also help in finding commonalities and differences across cancer driver genes. For example, one of the most commonly mutated families of proteins in cancer is receptor tyrosine kinases (RTKs). Among all of them, there are two particular subfamilies that are recurrently targeted by mutations: ERBB and FGFR. The first family includes EGFR, which is mutated in >4% of all cancers, as well as ERBB2 (2%), ERBB3 (2%) and ERBB4 (4%). Our method identified PPI interfaces in the first two members of this family, EGFR and ERBB2, as strongly enriched in mutations. These interfaces correspond to similar region of both proteins, as they both mediate dimerizations or interactions with other proteins of the ERBB family (Fig E). Remarkably, the interface responsible for the interaction with their ligands, such as EGF, is very rarely affected by mutations.

In the case of FGFR proteins, even though they are also RTKs, the mutation pattern is different, with two proteins, FGFR2 and FGFR3, having strong enrichments of mutations in the region involved in ligand binding, and none of them altering the homo- or heterodimerization interface.


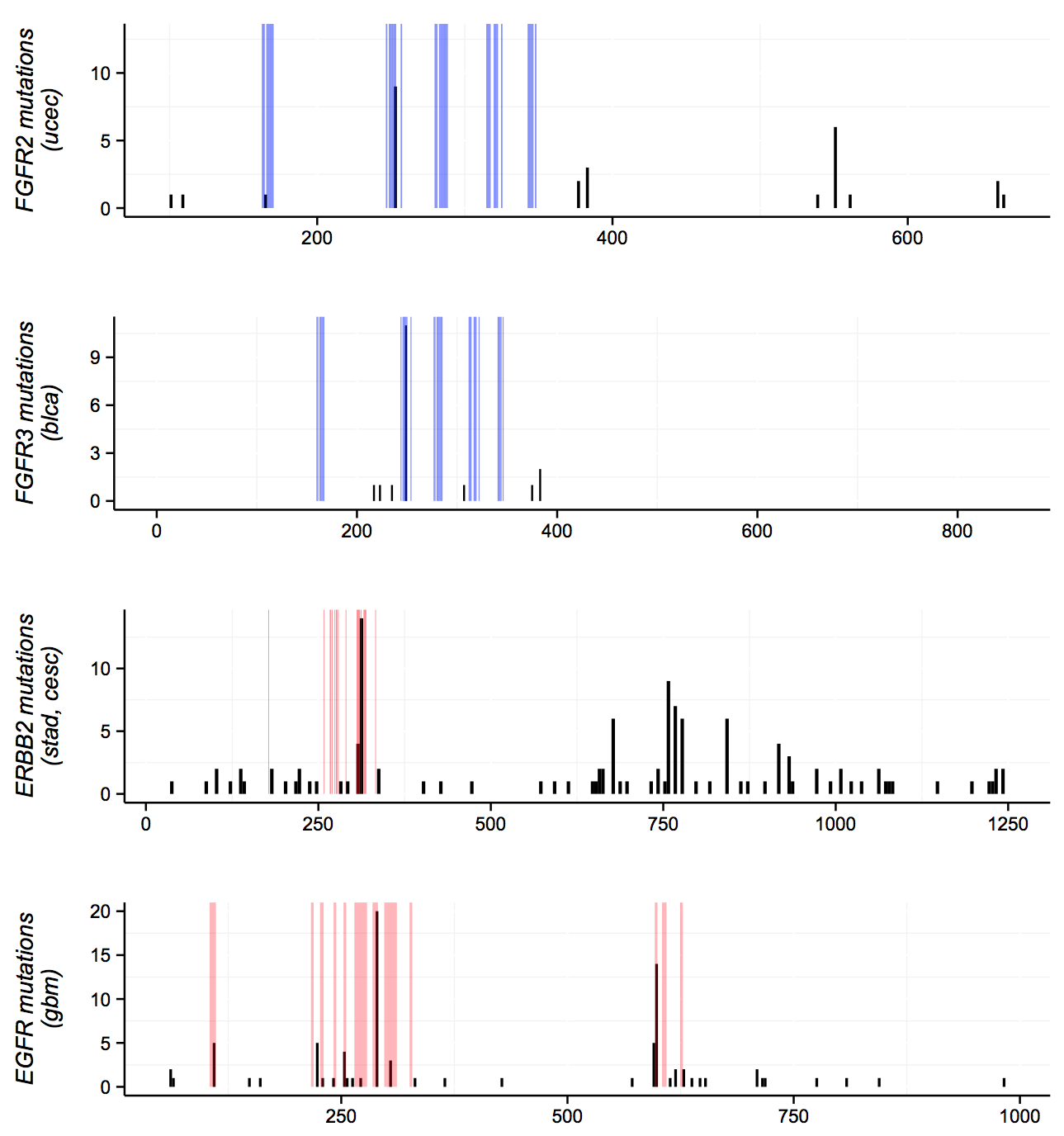


**Figure N – Mutation patterns in different receptor tyrosine kinases**. The histograms show the mutation frequencies (y axis) of different receptor tyrosine kinases across the protein (x axis). From top to bottom the proteins are FGFR2, FGFR3, ERBB2 and EGFR. Note also that each signal is tissue specific. The shaded areas indicate either residues involved in ligand binding (blue) or in protein homo- or heterodimerization (red).

Finally, we used other datasets from the same samples to gain insights into the consequences of the mutations in the different interfaces identified by e-Driver. Specifically, we downloaded the expression profiles of over 400 different proteins from 4,542 TCGA samples. These were measured by RPPA and can be downloaded from The Cancer Proteome Atlas^28^ and the TCGA data portal. Next, we compared the expression of the different proteins in three subgroups: (I) patients with mutations in the driver interfaces, (II) patients with mutations in other regions of the same protein and (III) patients with no mutations in that protein. We compared the expression levels using a Wilcoxon test and identified those proteins that were differentially expressed in group (I) when compared to both, group (II) and group (III). In order to limit the number of false positives caused by the intrinsic variability in protein expression across different tissues, we decided to do this particular analysis only for tissue-specific datasets. The direct consequence of this is that we could only study interfaces with high number of mutations (there is no proteomics data for all the TCGA samples yet), particularly EGFR and TP53. Data for EGFR is shown in Fig E and explained in the main text; here we show the results for TP53. Remarkably, we observed that there are 8 cancer types where samples with mutations in the dimerization and DNA interaction interface of TP53 have consistently higher levels of this protein than the other two subgroups. This highlights the importance of adding structural and functional context to the mutations instead of limiting our analysis to just the gene where the mutation occurs: there is no such thing as a TP53-driven tumor, each mutation in TP53 can have different consequences depending on the specific region that it affects.


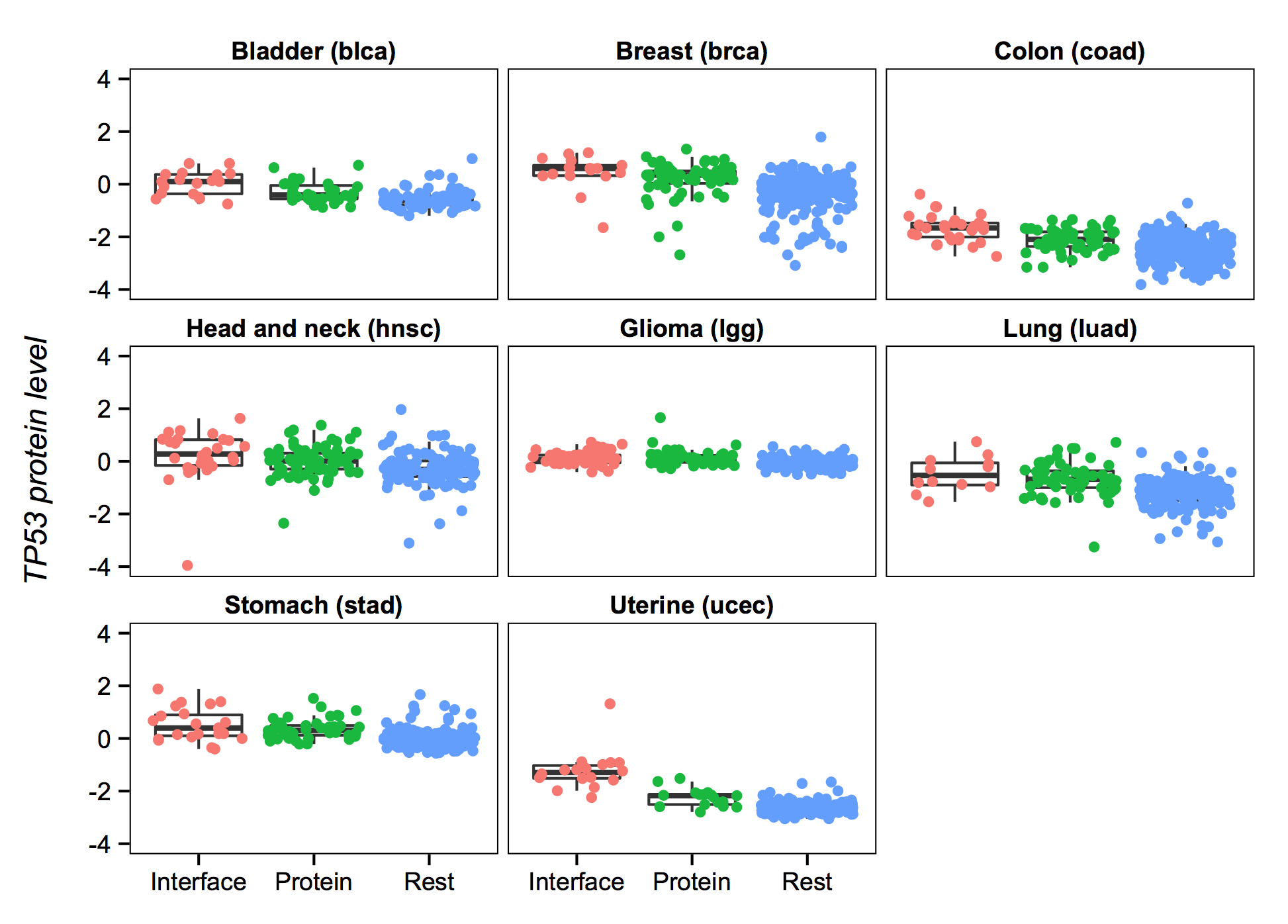
 **Figure O – TP53 protein levels change depending on the specific region mutated**. The y-axis shows the protein levels of TP53 in each of the 8 different cancer types where we found changes caused by mutations in the dimerization interface of TP53. Each dot represents a TCGA sample, and these are grouped and colored depending on where the mutation in TP53 is located: red for the interface, green for other regions of TP53 and blue for no TP53 mutations. In all cancer types there are statistically significant differences between the interface and the other two groups, except for “lgg”, “luad” and “stad”, where we did not find differences between the interface and protein groups.

**6 – Estimating the number of false positives**

Finally, in order to estimate the rate of false positives in our cancer driver predictions due to random clusters of mutations, we randomized the data in two different ways, repeated the analysis with the randomized data 100 times in each case, and calculated the number of interfaces identified by e-Driver in each simulation (FDR < 0.05).

In the first case we randomized the position of the mutations within each protein. This randomization keeps the number of mutations per protein constant, but it doesn’t necessarily keep the number of mutations within the structurally covered regions. In other words, if a protein that is 300 aminoacids long, with the region between 100 and 200 covered by a structure, originally had 5 mutations, all of them within the structure (i.e. between positions 100 and 200), after the randomization process the protein will still have the 5 mutations, but the region within 100 and 200 could have less. As shown in Fig P, the number of genes identified in this case was never above 2, and usually 0. In the second case, the randomization procedure consisted of altering the positions of the interfaces, while keeping the other variables (most importantly the position of the mutations and their frequencies) constant. In this case, the number of genes identified ranged between 20 and 24.

**Figure P – Estimation of the false positive rate.** These histograms show the number of genes identified in each randomization experiment, with the top histogram being the randomization of the mutation positions across the whole protein and the bottom histogram the randomization of the interface positions. In both cases the result in the original dataset (i.e. the 103 genes that we identified) is shown with an arrow for reference.

**7 - References**

1. Cancer Genome Atlas Research, N. *et al.* The Cancer Genome Atlas Pan-Cancer analysis project. *Nat Genet* **45**, 1113-20 (2013).

2. Lawrence, M.S. *et al.* Mutational heterogeneity in cancer and the search for new cancer-associated genes. *Nature* **499**, 214-8 (2013).

3. Altschul, S., Gish, W., Miller, W., Myers, E. & Lipman, D. Basic local alignment search tool. *J Mol Biol* **215**, 403-410 (1990).

4. Berman, H. *et al.* The Protein Data Bank. *Nucleic Acids Res* **28**, 235-242 (2000).

5. Flicek, P. *et al.* Ensembl 2012. *Nucleic Acids Res* **40**, D84-90 (2012).

6. Tamborero, D. *et al.* Comprehensive identification of mutational cancer driver genes across 12 tumor types. *Sci Rep* **3**, 2650 (2013).

7. Iakoucheva, L.M., Brown, C.J., Lawson, J.D., Obradović, Z. & Dunker, A.K. Intrinsic Disorder in Cell-signaling and Cancer-associated Proteins. *Journal of Molecular Biology* **323**, 573-584 (2002).

8. Shoemaker, B.A. *et al.* Inferred Biomolecular Interaction Server--a web server to analyze and predict protein interacting partners and binding sites. *Nucleic Acids Res* **38**, D518-24 (2010).

9. Ponstingl, H., Henrick, K. & Thornton, J.M. Discriminating between homodimeric and monomeric proteins in the crystalline state. *Proteins-Structure Function and Bioinformatics* **41**, 47-57 (2000).

10. Krissinel, E. & Henrick, K. Inference of macromolecular assemblies from crystalline state. *J Mol Biol* **372**, 774-97 (2007).

11. Zhang, Q.C. *et al.* Structure-based prediction of protein-protein interactions on a genome-wide scale. *Nature* **490**, 556-60 (2012).

12. Mosca, R., Ceol, A. & Aloy, P. Interactome3D: adding structural details to protein networks. *Nat Methods* **10**, 47-53 (2013).

13. Keshava Prasad, T.S. *et al.* Human Protein Reference Database--2009 update. *Nucleic Acids Res* **37**, D767-72 (2009).

14. Chatr-Aryamontri, A. *et al.* The BioGRID interaction database: 2015 update. *Nucleic Acids Res* **43**, D470-8 (2015).

15. Franceschini, A. *et al.* STRING v9.1: protein-protein interaction networks, with increased coverage and integration. *Nucleic Acids Res* **41**, D808-15 (2013).

16. Lee, I. *et al.* A single gene network accurately predicts phenotypic effects of gene perturbation in Caenorhabditis elegans. *Nat Genet* **40**, 181-8 (2008).

17. Aranda, B. *et al.* PSICQUIC and PSISCORE: accessing and scoring molecular interactions. *Nat Methods* **8**, 528-9 (2011).

18. Rolland, T. *et al.* A proteome-scale map of the human interactome network. *Cell* **159**, 1212-26 (2014).

19. Kotlyar, M. *et al.* In silico prediction of physical protein interactions and characterization of interactome orphans. *Nat Methods* **12**, 79-84 (2015).

20. Magger, O., Waldman, Y.Y., Ruppin, E. & Sharan, R. Enhancing the prioritization of disease-causing genes through tissue specific protein interaction networks. *PLoS Comput Biol* **8**, e1002690 (2012).

21. Lage, K. *et al.* A human phenome-interactome network of protein complexes implicated in genetic disorders. *Nat Biotechnol* **25**, 309-16 (2007).

22. Vanunu, O., Magger, O., Ruppin, E., Shlomi, T. & Sharan, R. Associating genes and protein complexes with disease via network propagation. *PLoS Comput Biol* **6**, e1000641 (2010).

23. Vidal, M., Cusick, M.E. & Barabasi, A.L. Interactome networks and human disease. *Cell* **144**, 986-98 (2011).

24. Kohler, S., Bauer, S., Horn, D. & Robinson, P.N. Walking the interactome for prioritization of candidate disease genes. *Am J Hum Genet* **82**, 949-58 (2008).

25. Jia, P. & Zhao, Z. VarWalker: personalized mutation network analysis of putative cancer genes from next-generation sequencing data. *PLoS Comput Biol* **10**, e1003460 (2014).

26. Jerby-Arnon, L. *et al.* Predicting cancer-specific vulnerability via data-driven detection of synthetic lethality. *Cell* **158**, 1199-209 (2014).

27. Hofree, M., Shen, J.P., Carter, H., Gross, A. & Ideker, T. Network-based stratification of tumor mutations. *Nat Methods* **10**, 1108-15 (2013).

28. Li, J. *et al.* TCPA: a resource for cancer functional proteomics data. *Nat Methods* **10**, 1046-7 (2013).
